# Enhancing CO_2_ emissions prediction for electric vehicles using Greylag Goose Optimization and machine learning

**DOI:** 10.1038/s41598-025-99472-0

**Published:** 2025-05-13

**Authors:** Ahmed El-Sayed Saqr, Mohamed S. Saraya, El-Sayed M. El-Kenawy

**Affiliations:** 1 Computer Engineering and Control Systems Department, Faculty of Engineering, Mansoura University, Mansoura, 35516 Egypt; 2 Department of Communications and Electronics, Delta Higher Institute of Engineering and Technology, Mansoura, 35111 Egypt

**Keywords:** $\hbox {CO}_2$ emissions prediction, Electric vehicles (EVs), Greylag Goose Optimization (GGO), Multi-Layer Perceptron (MLP), Sustainable transportation, Data processing, Machine learning

## Abstract

Electric vehicle (EV) $\hbox {CO}_2$ emissions should be predicted and mitigated, which requires lowering EV emissions in line with global sustainability goals. Such accurate forecasting supports policymakers and other industry stakeholders make marketing decisions to reduce environmental impacts and optimize resource utilization. In this research, a novel Greylag Goose Optimization (GGO) algorithm is integrated with a Multi-Layer Perceptron (MLP) model to improve $\hbox {CO}_2$ emissions prediction. Finally, the study does a comparative analysis with some established optimization algorithms in hyperparameter tuning regarding an improved accuracy model. In addition, statistical analyses such as ANOVA, sensitivity analysis, and T-test were used to substantiate performance differentiation between models. For the optimal model, the GGO-optimized MLP significantly outperformed baseline models and other optimization techniques, having minimum error metrics such as correlation coefficient and RMSE and an MSE of $4.72 \times 10^{-7}$. As a result, the emissions forecast is very reliable. The proposed approach provides actionable insights for environmental policies, EV adoption strategies, and infrastructure planning. The model enables stakeholders to achieve climate objectives, optimize EV charging systems and foster the creation of sustainable transportation systems, as said accurate emissions estimates are enabled.

## Introduction

$\hbox {CO}_2$ is a significant greenhouse gas contributing to the greenhouse effect and climate change. It causes global warming and climatic interferences, which are primarily driven by $\hbox {CO}_2$ and other greenhouse gases^1,2^. Human activities account for approximately 98% of $\hbox {CO}_2$ emissions, primarily arising from the combustion of fossil fuels for electrical power, transportation, and various industrial uses^3–5^. The rapid growth of industries and urbanization over the last century has exacerbated the depth of $\hbox {CO}_2$ in the environment, leading to environmental changes such as rising sea levels, increased abnormal weather events, reduced species diversity, and ecosystem conflicts^6–8^.

A major contributor to $\hbox {CO}_2$ emissions is the transportation sector, particularly from conventional Internal Combustion Engine (ICE) vehicles. Reducing emissions from this sector is essential to meet global climate goals and emissions reduction targets^9–11^. As a response, the adoption of Electric Vehicles (EVs), which generate almost no direct $\hbox {CO}_2$ emissions, is increasing. This shift towards EVs is aligned with the global effort to combat anthropogenic greenhouse gas emissions and promote sustainable resource utilization^12–14^. However, it is also important to consider the indirect $\hbox {CO}_2$ emissions associated with EVs, such as those resulting from the energy sources used for charging.

With the increasing adoption of EVs, the need for accurate models predicting $\hbox {CO}_2$ emissions has become more pressing. ICE vehicles emit $\hbox {CO}_2$ directly from fuel combustion, while EVs indirectly contribute to emissions based on the type of electricity used for charging^15–17^. When renewable energy sources such as wind and solar are used for charging, the $\hbox {CO}_2$ emissions are relatively low. However, emissions are higher when electricity is generated from fossil fuels like coal and natural gas^18–20^. Therefore, accurate predictions of $\hbox {CO}_2$ emissions are critical to assess the role of EVs in reducing greenhouse gases, particularly in regions with varying energy sources^21–23^.

Such predictions are essential for policymakers, utility providers, and environmental stakeholders. They can use the predictions to design strategies for scaling up the reduction of $\hbox {CO}_2$ emissions linked to EVs. For example, accurate emission forecasts could help develop incentives for renewable-based EV charging infrastructure, thereby enhancing the benefits of EV adoption^24–26^. Furthermore, emission predictions are valuable for regional and urban planning, assisting in determining where EVs would be most effective in reducing environmental impacts^27–29^.

From the consumer’s perspective, providing emission forecasts can increase the attractiveness of EVs by helping individuals understand the societal benefits of their decisions^30–32^. These predictions also assist the transportation and automotive industries in improving the efficiency of EV systems and charging stations, further contributing to $\hbox {CO}_2$ reduction. Forecasting $\hbox {CO}_2$ emissions is inherently challenging due to the uncertainty and non-linearity of the factors involved, especially when considering EVs^33–35^.

The development of EVs is crucial for advancing a low-carbon, sustainable economy. When powered by renewable energy sources, EVs can significantly reduce global $\hbox {CO}_2$ emissions^36^. The transportation sector is a major emitter of greenhouse gases, and as such, it has become a key focus for countries working towards fulfilling the targets set by the Paris Agreement and other climate frameworks. Additionally, EVs offer substantial air quality benefits, particularly in high-traffic areas, as they do not emit harmful pollutants such as NOx and particulates. This aligns with the goals of sustainable development, which aim to improve both environmental and public health outcomes^37,38^.

However, achieving these benefits is contingent upon reliable $\hbox {CO}_2$ forecasting. While direct emissions are relatively easier to measure, accurate prediction models for indirect emissions from EVs are critical for tracking their carbon footprint and formulating effective strategies to maximize their environmental benefits. These models can assist policymakers and stakeholders in designing cleaner EV charging systems and integrating renewable energy sources into the grid^39,40^. Furthermore, they can help identify optimal areas for EV adoption, reducing the negative environmental impacts associated with vehicle emissions in the long term.

In response to these challenges, this research proposes the integration of the Greylag Goose Optimization (GGO) algorithm with a Multi-Layer Perceptron (MLP) model to improve $\hbox {CO}_2$ emission predictions. GGO is a novel metaheuristic optimization algorithm inspired by the migratory and social behavior of Greylag geese. Unlike traditional optimization algorithms such as Particle Swarm Optimization (PSO) and Genetic Algorithm (GA), GGO dynamically balances exploration and exploitation by dividing the search agents into distinct groups. While PSO is computationally efficient, it often struggles with maintaining diversity in the search space and can prematurely converge in complex, multimodal optimization problems^41^. On the other hand, GA relies on genetic operators like mutation and crossover, which enhance exploration but are computationally expensive and require extensive parameter tuning^42^.

GGO distinguishes itself through adaptive intelligence mechanisms, inspired by the decision-making processes of geese during migration, which enable it to dynamically adjust its search strategies based on the problem landscape. This reduces stagnation and improves convergence efficiency. Moreover, GGO’s structured approach to exploration and exploitation helps it deal with complex, nonlinear data more effectively. Comparative results from this study demonstrate that GGO outperforms both PSO and GA in terms of error metrics and convergence rates, making it particularly well-suited for optimizing the hyperparameters of the MLP model in this context^43^.

To ensure clarity and consistency throughout this paper, the following Table 1 provides a list of symbols and abbreviations used. This nomenclature helps in understanding the terminology related to optimization algorithms, machine learning models, and performance evaluation metrics.

**Table 1:** List of symbols and abbreviations used in this paper.

Symbol/abbreviation	Description
$\hbox {CO}_2$	Carbon dioxide
EV	Electric vehicle
ICE	Internal Combustion Engine
GGO	Greylag Goose Optimization
BER	Binary Earth Radius Optimizer
GWO	Grey Wolf Optimizer
WWPA	Waterwheel Plant Algorithm
HHO	Harris Hawks Optimizer
PSO	Particle Swarm Optimization
JAYA	Jaya Optimizer
DTO	Dipper throated optimization
GA	Genetic Algorithm
SFS	Stochastic Fractal Search
WOA	Whale Optimization Algorithm
MLP	Multi-Layer Perceptron
K-NN	K-nearest neighbors
SVR	Support vector regression
MSE	Mean squared error
RMSE	Root mean squared error
MAE	Mean absolute error
*r*	Pearson correlation coefficient
$\hbox {R}^2$	Coefficient of determination
RRMSE	Relative root mean squared error
NSE	Nash-sutcliffe efficiency
WI	Willmott’s Index of Agreement

The contributions of this study are as follows:

1. *Application of GGO for Hyperparameter Optimization:* This research develops a novel method for improving the accuracy of $\hbox {CO}_2$ emissions models in the EV context by using the GGO algorithm to tune the MLP model’s hyperparameters.
2. *Comparative Analysis of Optimization Algorithms:* The study provides a comprehensive comparison between various optimization algorithms, including GGO, BER, GWO, WWPA, HHO, PSO, JAYA, DTO, GA, SFS, and WOA, to evaluate their performance in optimizing the MLP model’s hyperparameters.
3. *Improved* $\hbox {CO}_2$ *Emission Prediction for EVs:* The proposed MLP model captures the specific characteristics of EV-associated emissions, making it more effective and robust for emission estimation tasks.
4. *Scalable Solution for Environmental Policy and Industry Applications:* The developed model offers valuable insights for policymakers, urban planners, and industry stakeholders, helping them make informed decisions about EV adoption, infrastructure development, and emissions reduction strategies.

In conclusion, this paper demonstrates the use of GGO and MLP to develop an efficient model for estimating $\hbox {CO}_2$ emissions in the EV industry, offering a flexible and highly accurate approach that supports sustainable transportation solutions.

## Literature review

Known as the ‘Going Green’ campaign, the search for a world free from carbon emissions has inspired several advancements in energy and transport. These industries contribute highly to global $\hbox {CO}_2$ emissions, so implementing strategies for managing gas emissions and improving energy utilization is necessary. This literature review synthesizes existing literature and recent advancements in emissions, energy use, and critical indicators essential for sustainable mobility and urban planning.

Due to the rapid evolution of renewable energies and the rising demand for EVs and HEVs, research has shifted toward improving Multi-Energy Systems (MESs) and vehicle energy efficiency. The use of complex approaches such as deep learning, multi-objective optimization, and hybrid algorithms is essential to manage the dynamics of energy management and emission control. These approaches advance the frontiers of knowledge and provide solutions to complex issues in sustainable cities, photovoltaic systems, and improving ground vehicle performance.

One sector of focus is intelligent zero-carbon multi-energy systems and emission scheduling models. For instance,^44^ introduced a deep learning and optimization methodology for operational scheduling of zero-carbon multi-energy systems (ZCMES) in virtual power plants. This model incorporates new capture technologies, time-varying EV charging and discharging specifications, and clean energy market plans to improve the system’s robustness while reducing emissions. Applying statistics of previous occurrences in Arizona, the study integrated prognostics with the usage of GRU-BiLSTM for scenario generation, along with a robust stochastic method for decision-making, which proved effective. The evaluation revealed a 10.74% improvement in day-ahead scheduling costs and a 36% decrease in conventional emissions, indicating the model’s applicability as a potential blueprint for energy policy frameworks and zero-carbon cities. Thus, this work demonstrates that future optimization algorithms must be incorporated to achieve systemic and sustainable effects in the energy sector.

It also shows that understanding the efficiency of EVs compared to ICE vehicles is becoming critical for evaluating the actual environmental advantages of EVs. In^45^, researchers developed a comprehensive model to evaluate EV range capabilities across urban routes using five critical driving cycles: uniform movement, increasing and decreasing velocity, constant velocity, uphill movement, and downhill movement. This approach captured driving behaviors from aggressive to moderate, providing insights into fuel savings and emissions under different urban conditions. By emulating these dynamic processes, the model provided critical data for policymakers and urban planners to best utilize the growing trend of EVs within the current infrastructure. These analyses also closed discrepancies between theoretical fuel efficiency and absolute emissions values, ensuring that the impact of EVs on urban environments is accurately reflected in planning and legislation.

Another significant stream of current research focuses on correctly identifying optimal locations for EV charging stations in electric distribution systems (EDS), particularly emphasizing renewable energy sources. In the study by^46^, a new model was developed to determine effective locations for EV charging stations regarding environmental and operational impact. The model utilized mixed-integer linear programming (MILP) methodologies to address uncertainties and incorporate time-varying energy demand and supply profiles alongside heterogeneous EV charging patterns. Conducted on a 24-node system, the proposed model demonstrated that the socio-economic benefits of EV integration can only be fully realized by incorporating renewable energy.

Optimizing HEVs is more complex than optimizing their ICEV counterparts due to their intricate powertrain topology. In^47^, these challenges were addressed using a co-optimization model that adjusts powertrain control parameters based on expected driving speeds. The solution maximized a two-objective function targeting fuel consumption reduction and emission minimization by applying a neural network-based model. Applied to Simulink data from a Toyota Prius Hybrid, the co-optimization strategy provided a 9.22% gain in fuel economy. This paper demonstrated how predictive driving data can facilitate real-time control adjustments in HEV powertrains, resulting in efficiency improvements and corresponding emission reductions. These findings lay the groundwork for the next generation of vehicles, which will feature higher responsiveness on the road.

If cities are to become sustainable, the electrification of mobility and road transport is inevitable. In^48^, an analytical model for altering vehicle energy consumption based on city traffic regulations was discussed. Using stochastic speed profiles and multiple linear regression, it investigated energy and emission outcomes in various traffic situations. When applied to realistic urban scenarios, the highlights have significantly impacted decision-making among urban planners and fleet managers by enabling effective traffic flow management and minimizing transport-related emissions. This research highlights the importance of predictive methodologies for modeling and controlling traffic and emissions in densely built urban areas to enhance sustainable transport systems.

AI and BI are increasingly used to prepare less carbon-dependent economic solutions and improve the reliability of emission predictions. The work by^49^ presented a novel Multi-Universe Quantum Harmony Search Algorithm-Dynamic Fuzzy System Ensemble (MUQHS-DMFSE) to predict carbon emissions with high accuracy, maintaining a mean absolute percentage error (MAPE) below 3.5%. A hybrid model integrating artificial intelligence, big data analytics, and fuzzy logic was developed and applied for emission prediction and combined with DEA CCR and BCC models to evaluate the low-carbon efficiency of the economy. The study’s conclusions provide directions for building an energy structure that incorporates clean energy and highlight the role of sophisticated analytical methods in formulating sustainable economic plans and emission control measures.

In^50^, the authors proposed applying deep neural networks (DNNs) to improve HEV powertrain design by predicting $\hbox {CO}_2$ emissions relative to diverse powertrain parameters. The model achieved a simulation accuracy of 91.4%. Such predictive features enable the identification of the most efficient HEV powertrain arrangements, thereby increasing fuel economy and reducing emissions. This work illustrates the value of machine learning approaches in vehicle design and encourages the development of HEVs that combine excellent performance with strict adherence to environmental standards.

The $\hbox {CO}_2$ emissions of HEVs in^51^ were investigated using the gradient boostiGradientithm on Toronto and Beijing’s vehicles’ kinematics data. The emissions were tested during the realistic performance of the vehicle under three cycles, and it was observed that a substantial reduction in the $\hbox {CO}_2$, CO, and NOx emissions was achieved. These outcomes produce helpful knowledge of traffic conditions and drivers’ behavior, which helps create concrete emissions estimations for HEVs and their control for urban areas. Safe traffic flow and low emission control recommendations are the pro-environmental driving behaviors that policies should embrace.

Integrated systems with Plug-in Electric Vehicles require schemes and approaches for efficiently governing usage and emissions off-a multi-objective performance model that can maximize profit while minimizing the $\hbox {CO}_{{2}}$ emissions has been presented by^52^. Thus, this model of PEV was designed to identify the best charging and discharging patterns from the overall demand by applying the MGSO algorithm and reducing the emission intensity level up to 75%. Outcomes during the simulations covered the model’s efficacy in urban areas regarding energy efficiency utilization and emissions levels. Such results imply the necessity of an integrated charging model and elaborated mechanisms to optimize the efficiency of utilization of such types of automobile vehicles as EVs and PEVs.

The control of $\hbox {CO}_2$ emissions at intersections, especially in mixed traffic, was discussed in^53^. The authors presented an Optimal Signal Timing model targeting the minimization of vehicle delays, stopping rates, and emissions. The research demonstrated how optimized signal timings could regulate traffic and reduce emissions in detailed case studies. This study provides practical insights for urban planners and traffic engineers, suggesting that signal optimization can be an effective strategy for enhancing mobility while reducing the harmful effects of vehicle emissions in urban areas.

Stochastic Fractal Search (SFS) is a new metaheuristic algorithm based on the formation of fractals in nature^54^. The SFS algorithm is enhanced to possess diversity capability, and the Fitness-Distance Balance (FDB) operator is incorporated into SFS, producing the FDB-SFS algorithm. Superiority over 39 competing algorithms was demonstrated across 89 test functions and five constrained engineering problems using experimental studies. Furthermore, in addition to the well-known method of interframe coding, we also apply intercolor coding and use it to encode the chrominance channel. Results show that the FDB-SFS is by far superior to competing algorithms in terms of robust search.

Inspired by Harris’ hawks cooperative hunting behavior, Harris Hawks Optimizer (HHO) is designed. HHO mimics dynamic chasing patterns and therefore adapts to a variety of scenarios across^55^. HHO was applied to 29 benchmark problems and several real-world engineering problems; HHO steadily provided competitive or promising results as compared to existing metaheuristics. Because it is made public, it can be adopted more broadly and used in more optimization tasks.

To handle optimization problems, the Waterwheel Plant Algorithm (WWPA) models the hunting behavior of the waterwheel plant as search agents^56^. In addition, evaluations of 23 objective functions demonstrated WWPA’s ability to exploit and explore intensively, which was useful for unimodal and multimodal problems. When compared with seven well-known metaheuristics, WWPA is shown to provide significantly better performance while maintaining a proximal balance between exploration and exploitation, thus underlying its potential applicability to real engineering problems.

DTO is a novel metaheuristic algorithm inspired by the rapid bowing movements of the dipper-throated bird^57^. Its efficiency was further confirmed in comparative tests against PSO, WOA, GWO, and GA on seven unimodal benchmark functions and statistical validation through ANOVA and Wilcoxon rank-sum tests. Experimental results of its application to feature selection tasks using UCI datasets showed that DTO has the capability to solve complex problems in the real world and outperform traditional feature selection algorithms in terms of performance.

Jaya algorithm is a simple, yet effective parameter-less metaheuristic. The Jaya algorithm optimizes with no parameter tuning but simply by iterative improvement of candidate solutions^58^. This approach is simple to implement and highly applicable. Due to its versatility and ease of use, the algorithm is a useful addition to the optimization toolkit, especially for practitioners that aim for easy-to-use and efficient solutions without parameter adjustment.

A novel approach is introduced based on the swarm behavior and the Al-Biruni Earth radius calculation method^59^ using the Al-Biruni Earth Radius (BER) optimization algorithm. Comparative analysis with state-of-the-art metaheuristics on the solutions of seven mathematical optimization problems and an engineering design task corroborates the effectiveness of BER in exploring the search space and a cursory bypass of local optima. The results show that it offers better performance and capability, making BER a robust tool for optimization challenges.

Table 2 summarizes the studies discussed, focusing on $\hbox {CO}_2$ emission reduction and prediction in sustainable transportation, energy management, and urban planning contexts. This table organizes each study’s key objectives, methodologies, and significant findings.

**Table 2:** Summary of literature review on $\hbox {CO}_2$ emission reduction and prediction models.

Reference	Objective	Methodology	Key findings
^44^	Develop scheduling model for zero-carbon multi-energy systems in virtual power plants	Deep learning (GRU-BiLSTM) and robust stochastic optimization based on Arizona data	Significant cost reduction (− 10.74%) and environmental emission decrease (− 36%)
^45^	Evaluate the efficiency of EVs versus ICE vehicles on urban routes	Driving cycles covering aggressive, normal, and moderate driving styles	Useful for environmental implications of EV use in urban environments
^46^	Integration of EV charging stations with renewable energy into electric distribution systems	Mixed-integer linear programming (MILP) applied on a 24-node system	Highlights renewable energy’s role in realizing the environmental benefits of EVs
^47^	Optimize HEV powertrain for fuel efficiency and emissions control	NN-based model for speed prediction and powertrain control optimization	Achieved additional 9.22% fuel savings on Toyota Prius Hybrid
^48^	Estimate EV energy consumption impact based on traffic regulation changes	Stochastic speed profiles and multiple linear regression for urban planning	Assists in traffic flow enhancement and emissions reduction in urban settings
^49^	Formulate low-carbon economic strategies using carbon emissions prediction	MUQHS-DMFSE model (multi-universe quantum harmony search in fuzzy system)	High accuracy (MAPE < 3.5%) with applications in sustainable economic policies
^50^	Design parameter prediction model for HEVs to achieve desired $\hbox {CO}_2$ emissions	Deep neural networks with the pipeline for correlations between powertrain characteristics	Simulation test accuracy over 91%; useful for environment-friendly HEV designs
^51^	Real-world $\hbox {CO}_2$ emissions assessment for HEVs in different regions	Gradient boosting algorithm based on Toronto and Beijing driving cycle data	Indicates large influence of traffic flow and driving behavior on HEV emissions
^52^	Energy management for local multi-energy systems with PEVs	Multi-objective optimization model with Modified Group Search Optimization (MGSO)	Optimizes energy use and emissions in urban multi-energy systems
^53^	Develop signal timing model to reduce emissions at intersections with mixed traffic	Optimal signal timing using case study comparisons of emissions and traffic flow	Demonstrates viability of optimized signal timing in urban traffic emission management
^54^	Improve the diversity and balance between exploration and exploitation in SFS	Developed the FDB-SFS algorithm by incorporating a Fitness-Distance Balance operator to mimic natural fractal behavior	Achieved superior search performance, reducing premature convergence and ranking first among 39 competing algorithms
^55^	Develop a novel optimization algorithm inspired by Harris’ hawks	Mimicked the cooperative and dynamic chasing behavior of Harris’ hawks using mathematical models	Provided competitive results on 29 benchmark problems and engineering applications, demonstrating high adaptability
^56^	Propose an optimization algorithm based on the waterwheel plant’s behavior	Modeled the waterwheel plant’s prey-hunting behavior as a stochastic optimization technique	Balanced exploration and exploitation effectively, outperforming seven competing algorithms on unimodal and multimodal problems
^57^	Introduce a metaheuristic inspired by the hunting behavior of the dipper-throated bird	Simulated rapid bowing movements for optimization and tested the algorithm on unimodal benchmark functions and real-world feature selection problems	Outperformed traditional algorithms (PSO, WOA, GWO, GA) in solving feature selection problems
^58^	Present a parameter-less optimization algorithm	Focused on iterative improvement of candidate solutions without the need for parameter tuning	Demonstrated versatility, simplicity, and broad applicability in solving optimization problems
^59^	Propose a novel algorithm inspired by Al-Biruni’s earth radius calculation method	Explored the search space based on swarm behavior and the Al-Biruni radius calculation technique	Avoided local optima and outperformed state-of-the-art algorithms on mathematical and engineering optimization problems

*Research Gap* Despite significant advancements in predictive modeling for $\hbox {CO}_2$ emissions in the transportation sector, several critical gaps remain unaddressed. Existing studies primarily focus on conventional Internal Combustion Engine (ICE) vehicles, neglecting the complexities associated with Electric Vehicles (EVs) and their indirect emissions from electricity generation. Although machine learning (ML) models have been employed to enhance emission predictions, their performance is heavily reliant on hyperparameter tuning, which remains a challenge due to the lack of efficient optimization techniques.

Furthermore, while traditional metaheuristic algorithms such as Particle Swarm Optimization (PSO), Genetic Algorithm (GA), and Grey Wolf Optimizer (GWO) have been utilized for parameter tuning in ML models, they exhibit limitations in balancing exploration and exploitation, often leading to suboptimal convergence and premature stagnation. Additionally, existing studies fail to comprehensively evaluate metaheuristic algorithms in optimizing emission prediction models, particularly in the context of EVs, where emission dynamics are influenced by energy sources and regional grid compositions.

To bridge this gap, this research introduces the Greylag Goose Optimization (GGO) algorithm as an innovative approach for optimizing Multi-Layer Perceptron (MLP) hyperparameters. By leveraging GGO’s adaptive intelligence and structured migration-based search mechanisms, the study aims to achieve superior prediction accuracy while mitigating premature convergence issues inherent in other metaheuristic techniques. This work further contributes by conducting a rigorous comparative analysis against established optimization methods and validating the proposed model using extensive statistical evaluations, including ANOVA and T-tests. The insights derived from this study provide actionable recommendations for policymakers and industry stakeholders in formulating effective EV adoption and emission reduction strategies.

## Dataset description and statistical analysis

### Dataset overview

For this analysis, we employed a substantial database containing data on fuel economy and projected $\hbox {CO}_2$ emissions of new LDVs in Canada from the year 2000 to 2022^60^. LDVs, which are cars, SUVs, and small trucks, account for a significant market share and thus are essential to analyze when estimating the transportation sector’s $\hbox {CO}_2$ emissions. The dataset includes records, each of which possesses information on an individual model and fuel consumption ratings under city and highway circumstances. A combined fuel economy rating considers 55 percent of city driving and 45 percent of highway use. Petrol and diesel consumption is measured in liters per 100 kilometers (L/100 km), with miles per gallon (mpg) equivalents outlined.

The computed average overall $\hbox {CO}_2$ emissions estimates are expressed in grams per kilometer (g/km) and rely on the vehicle’s fuel type and combined fuel consumption factor. This way, the dataset contains various fuels: regular gasoline, premium gasoline, diesel, ethanol (E85), and natural gas, allowing investigation into the differences in emissions between fuel types. Furthermore, the characteristics of a specific vehicle are provided, including the type of transmission (automatic, manual, continuously variable transmission), drive type (4WD/4X4, AWD), and the number of engines. These details are paradigms for understanding how various elements affect fuel consumption and emissions. Notably, the fuel consumption ratio for 2000-2022 has been recalculated to reflect the aforementioned current methodology and better reflect reality. However, this shift allows the dataset to compare trends in fuel utilization and $\hbox {CO}_2$ emissions over any period.

However, some things about the dataset are limited and potentially biased. The data is geographically constrained to vehicles in Canada and, therefore, cannot be generalized to other geography whose vehicle usage patterns, fuel compositions, and energy mixes differ from those in Canada. For instance, there is a wide disparity in the fraction of electricity that comes from renewable sources vs. fossil, and that directly affects the $\hbox {CO}_2$ emissions made by electric vehicles. The dataset also covers a period of temporal variability from 2000 to 2022, potentially missing newer technological advancements and more future forecasts on the adoption of EVs and more renewable energy usage.

Finally, the data set could not sufficiently capture the variability in $\hbox {CO}_2$ emissions from different driving conditions like severe weather, different traffic densities, and variation in urban driving versus rural driving. However, these factors can significantly impact fuel consumption and emissions, but they may not be fully represented in the dataset. For example, average emissions data can hide how much specific patterns like frequent stop-and-go traffic or aggressive driving affect fuel consumption.

Finally, while the dataset is rich with information regarding trends in $\hbox {CO}_2$ emissions versus time and among different vehicle types and fuel sources, there are geographic and temporal limitations and points of missing variability in the driving conditions captured in the data. Future studies could address these approaches’ limitations using data across multiple countries, with a greater variety of driving scenarios, and by including more detailed information regarding energy mix.

### Statistical summary

We also performed a statistical analysis on the principal parameters of measured fuel consumption using various driving cycles: city, highway, and combined, and calculated $\hbox {CO}_2$ emissions. The cross-referenced considerations mentioned above reference fuel consumption and emission values; still, significant variability exists due to car features. For descriptive assessment of the variables and to determine the degree of variation each variable possesses, we used measures like mean, median, standard deviation, and range.

- *Fuel Consumption (L/100 km):* The authors indicate that the average fuel consumption ratings vary depending on the type of driving, with city driving using more fuel than highway driving because of the various stop-and-go conditions within city limits. In this category, many models consumed a significantly larger amount of fuel per average kilometer, which is logical since higher weight requirements and greater power are needed to drive an SUV or a truck. Even for larger vehicles, the standard deviation for city and highway consumption is also higher, suggesting larger coefficients of variation for city and highway consumption across the models of larger vehicles.
- $\hbox {CO}_2$ *Emissions (g/km):* The emissions of $\hbox {CO}_2$ gas per kilometer are also variable depending on the vehicle available on the market. The low-emitting vehicles include small-sized, fuel-efficient cars, while the high-emitting vehicles include large, less fuel-efficient models with worse $\hbox {CO}_2$ emissions. Examining the data, we find that yearly average $\hbox {CO}_2$ emissions have gradually declined in newer models, suggesting that effective fuel economy and emissions mitigation measures have been implemented. The standard deviation of emissions showed that although several vehicles have improved, there is still significant scatter, particularly among high-output engines.
- *Transmission and Fuel Type:* The dataset features various forms of transmission, including automatic transmission systems, manual systems, and other unique forms such as CVT and AM transmission systems. Understanding such transmissions reveals that automatic transmissions with SS CVTs and those analyzed as CVTs generally reflect better fuel economy, with a preference towards recently manufactured cars. Regarding fuel types, most automobiles running on ethanol (E85) and CNG emit fewer emissions than gasoline or diesel, emphasizing the environmental advantages of alternative fuels.
- *Vehicle and Drive Type:* Looking at the type of vehicle and drive, it is possible to identify specific emissions and fuel consumption features. For instance, 4WD and AWD car models generally have relatively high fuel consumption rates due to their power arrangement processes, especially in urban traffic patterns. Front-wheel drive (FWD) cars, characteristic of most compact vehicles, exhibit better fuel economy than their rear-wheel-drive counterparts, further supporting the environmental benefits of small car usage within city environments.

### Data preprocessing

Comprehensive preprocessing procedures were conducted to improve data quality, temporal consistency, and upgradability to transform the obtained dataset into a format suitable for effective predictive modeling and exploratory data analysis. This paper mainly focuses on data preprocessing aspects for machine learning applications, as these determine the performance of models and the accuracy of forecasted outcomes. These general preparatory procedures involved addressing missing values, scaling continuous variables, and creating dummy variables from categorical predictors. Each step aimed to reduce data quality concerns and ensure compatibility with different machine learning algorithms.

- *Handling Missing Values:* The first step was handling missing values, which can disturb analyses and models. Depending on their location and frequency within the dataset, we handled missing values using different strategies:
  - *Continuous Variables:* For non-categorical measurements like fuel efficiency and $\hbox {CO}_2$ emissions in city, highway, and combined cycles, missing values were imputed using either the mean or median of all values, depending on the data’s kurtosis. The mean was used if the data distribution was approximately normal; otherwise, the median was used to minimize influence. This approach preserved data continuity without imposing artificial fluctuations.
  - *Categorical Variables:* Based on exploratory analyses and previous research, missing values in categorical predictors were replaced by the most frequent category within subsets of data, such as within the same model year, make, or model. This ensured that the imputation maintained data patterns and avoided biases that could distort the dataset.
  Imputation procedures were only applied to the training set, with similar transformations performed on the test set to avoid compromising the model.
- *Normalization and Standardization:* Standardizing continuous data was necessary to prevent discrepancies in scale that could affect certain algorithms. Two main scaling techniques were applied:
  - *Normalization:* Variables were standardized to a 0 to 1 range, enabling comparisons between vehicles from different models and manufacturing years. Normalization benefited variables like fuel consumption and $\hbox {CO}_2$ emissions, where scales varied widely.
  - *Standardization:* For some models, features were standardized to a mean of 0 and a standard deviation of 1. This ensured all continuous variables were weighted equally during model training, especially for algorithms relying on gradient-based optimization techniques.

As mentioned, the choice between normalization and standardization depended on the properties of the applied machine learning algorithms.

- *Encoding Categorical Variables:* Because many machine learning algorithms involve numerical inputs, categorical variables were transformed through the following techniques:
  - *One-Hot Encoding:* For features with multiple categories (e.g., the fuel type), one-hot encoding was applied without introducing an order hypothesis. This ensured that the contribution of each category to the overall $\hbox {CO}_2$ emissions was more democratized.
  - *Label Encoding:* Binary and few-category features were encoded by interpreting non-numerical features into a scaled form. Where ordinal relationships were not realistic, these features were converted into integers.
- *Feature Engineering and Interaction Terms:* Additional data were aggregated to obtain new variables. For example, the interaction between fuel type and engine size was calculated, as it could have curvilinear impacts on $\hbox {CO}_2$ emissions.
- *Outlier Detection and Treatment:* Outliers were defined using standard methods, including IQR and z-score approaches. Influence values were either Winsorized or excluded to ensure that the models focused on normal ranges rather than extreme outliers, which could disproportionately affect the results.
- *Splitting the Dataset:* The dataset was divided into three sets: training, validation, and test sets, with a ratio of 80:10:10, respectively. Stratified sampling was applied to ensure proper data splitting while retaining variety and homogeneity in vehicle types.

These preprocessing steps were vital for obtaining clean data, minimizing adverse external effects on the training process of models, and subsequently enhancing the precision of the predictive models for $\hbox {CO}_2$ emissions. They aid in evaluation and decision-making concerning environmental and transport policies.

### Exploratory analysis

Preprocessing and data visualization through EDA helped identify the dataset’s trends, relationships, and patterns. Key findings from the EDA include:

- *Trends Over Time:* The trend changes dramatically from 2000 to 2022 for both fuel consumption and $\hbox {CO}_2$, where the latter decreases due to better vehicle standards and technologies. High emission standards and market forces that compel consumers to look for fuel-efficient cars have pushed producers to improve technology and construct better fuel-saving engines. Hybrid and electrical automobiles are relatively rare in the dataset but contribute to this decline since they produce far fewer emissions most of the time compared to conventional automobiles.
- *Fuel Type and Emissions Relationship:* Cars that run on alcohol (E85) and natural gas (CNG) produce relatively low levels of $\hbox {CO}_2$ emissions compared to cars powered by gasoline or diesel. This trend underlines the potential advantages of transitioning to other forms of fuel to reduce emissions. There are occasional marginal improvements in efficiency for vehicles using premium gasoline. Still, these are minor, indicating that fuel plays a significant role in emissions, primarily when using lower-carbon fuels.
- *Transmission Type Influence:* Newer automatic transmission technologies like CVTs and automated manuals are strongly associated with better fuel economy. These transmissions enable effective rate matching or, on average, better gear selection, which promotes fuel efficiency and reduces $\hbox {CO}_2$ emissions. This trend is most apparent in compact and midsize cars, where carmakers use CVT technology to meet corporate fuel economy standards. While automatics were historically less efficient than manuals, automatic transmissions have gained prominence recently as new model cars come with fewer manual transmission systems.
- *City vs. Highway Consumption Patterns:* Fuel consumption in city driving patterns is higher than in highway driving due to frequent acceleration and deceleration in congested traffic. Combined fuel economy ratings show that high-achieving cars tend to demonstrate a more conspicuous disparity between city and highway consumption levels due to the weight and size of the engine used. Conversely, small cars exhibit smaller disparities in city and highway consumption due to fuel efficiency in urban central use.

**Fig. 1:**
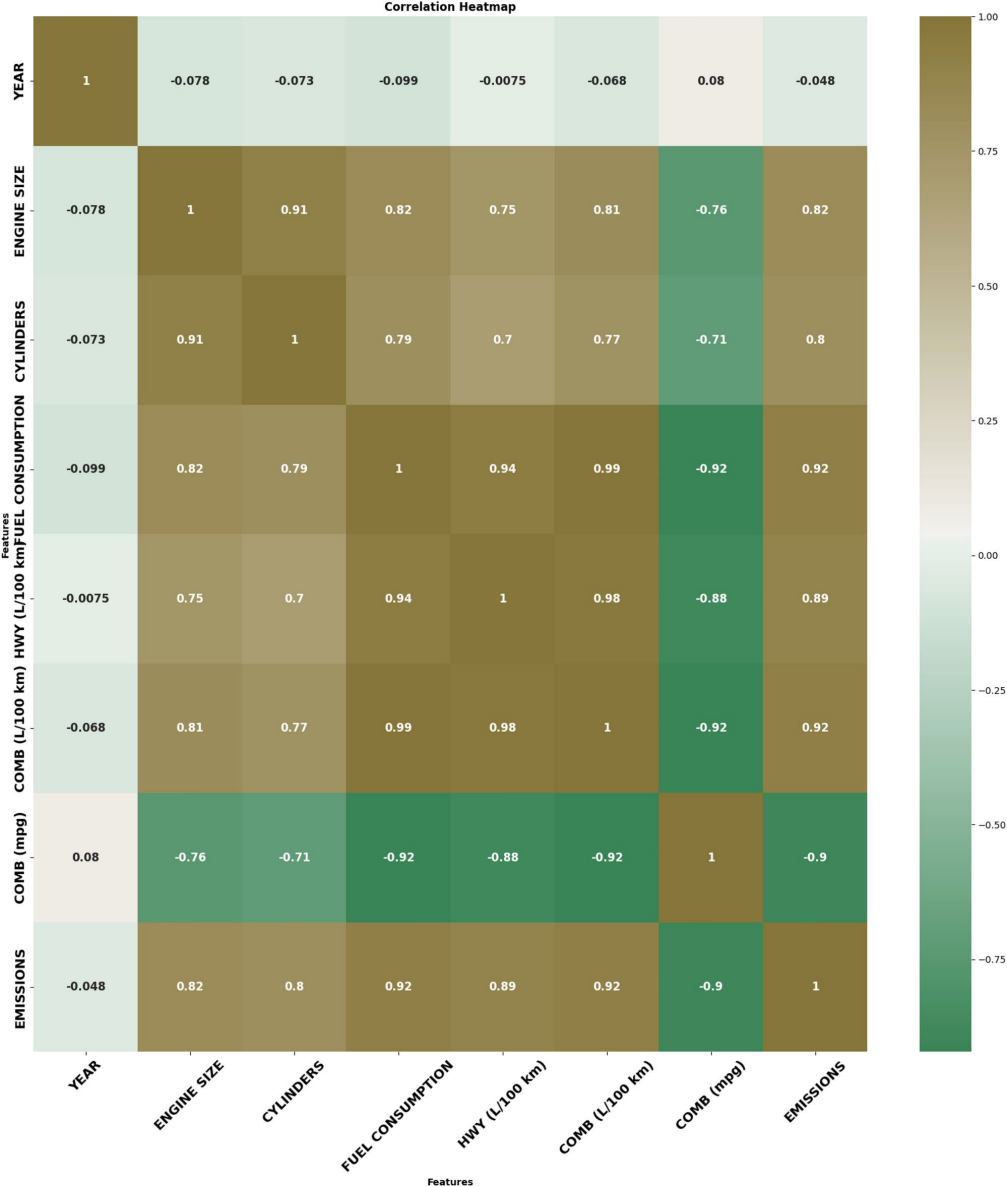
Correlation matrix for key variables.

Figure 1 illustrates the correlation between critical variables in the dataset, including engine size, number of cylinders, fuel consumption (city and highway), combined fuel economy (in L/100 km and mpg), and $\hbox {CO}_2$ emissions. The heatmap provides a clear visual representation of the strength and direction of relationships among these variables. Strong positive correlations are shown between engine size, cylinders, and fuel consumption metrics, indicating that larger engines and more cylinders are associated with higher fuel consumption and emissions. Conversely, a negative correlation exists between combined fuel economy (mpg) and emissions, highlighting that vehicles with higher fuel efficiency emit less $\hbox {CO}_2$.

**Fig. 2:**
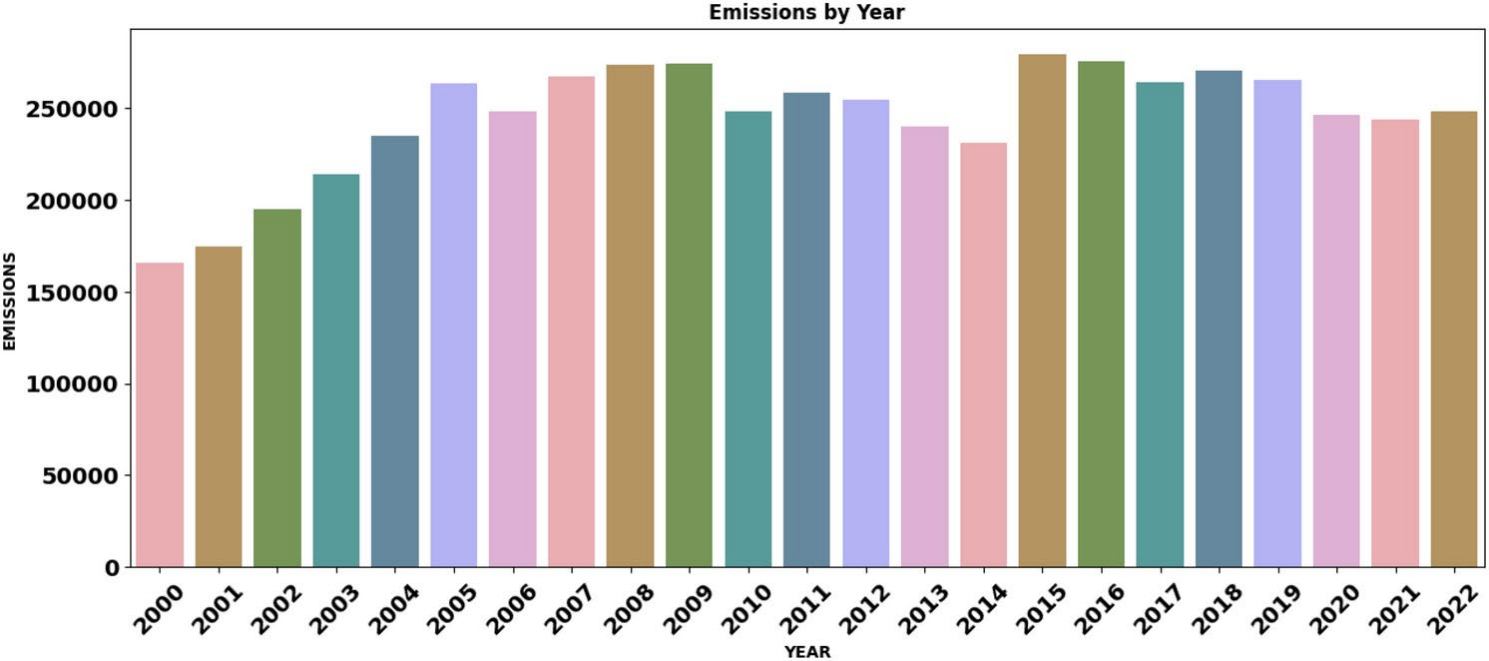
$\hbox {CO}_2$ emissions over time (2000–2022).

Figure 2 represents the total $\hbox {CO}_2$ emissions produced by vehicles over time from 2000 to 2022. The bar chart provides insights into the changes in emissions levels across different years, which may reflect shifts in vehicle technology, regulatory standards, and fuel consumption patterns. Peaks observed during specific years may indicate higher emissions due to the production and sales of less fuel-efficient vehicles. At the same time, declines may correspond to advances in emission control technologies and increased adoption of fuel-efficient or alternative-fuel vehicles.

**Fig. 3:**
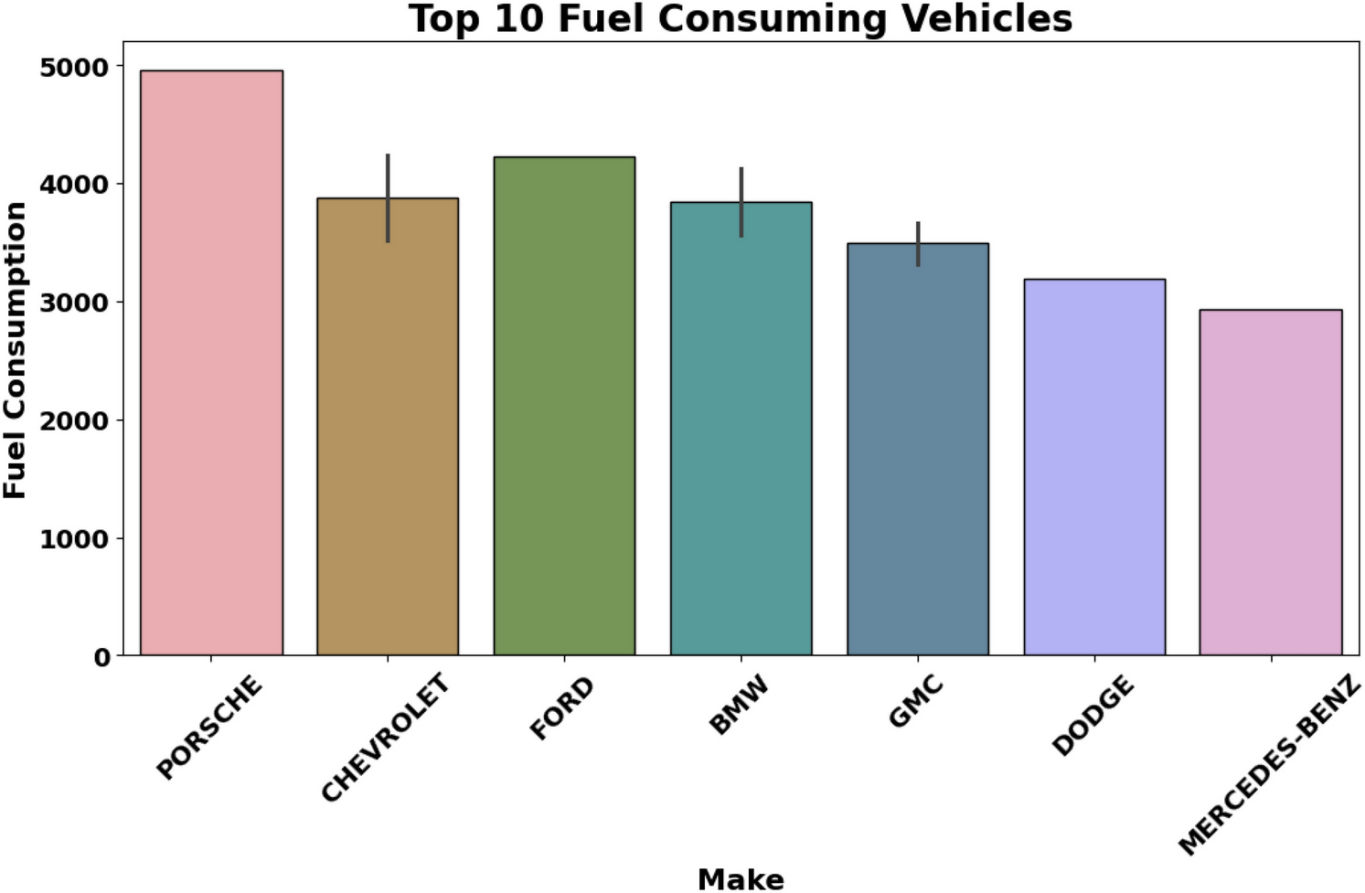
Fuel consumption by vehicle manufacturer.

Figure 3 represents manufacturers with the average fuel consumption per 100 km in liters. This feature illustrates how some car makers, like Porsche, have a comparatively larger average fuel consumption, while others, like Mercedes-Benz, have comparatively more minor average fuel consumption. This visualization helps identify manufacturers significantly contributing to fuel consumption and $\hbox {CO}_2$ emissions. It can also refer to the comparison of various types of vehicles manufactured by different Audi and BMW plants: for example, sports cars, sedans, hybrids, etc.

**Fig. 4:**
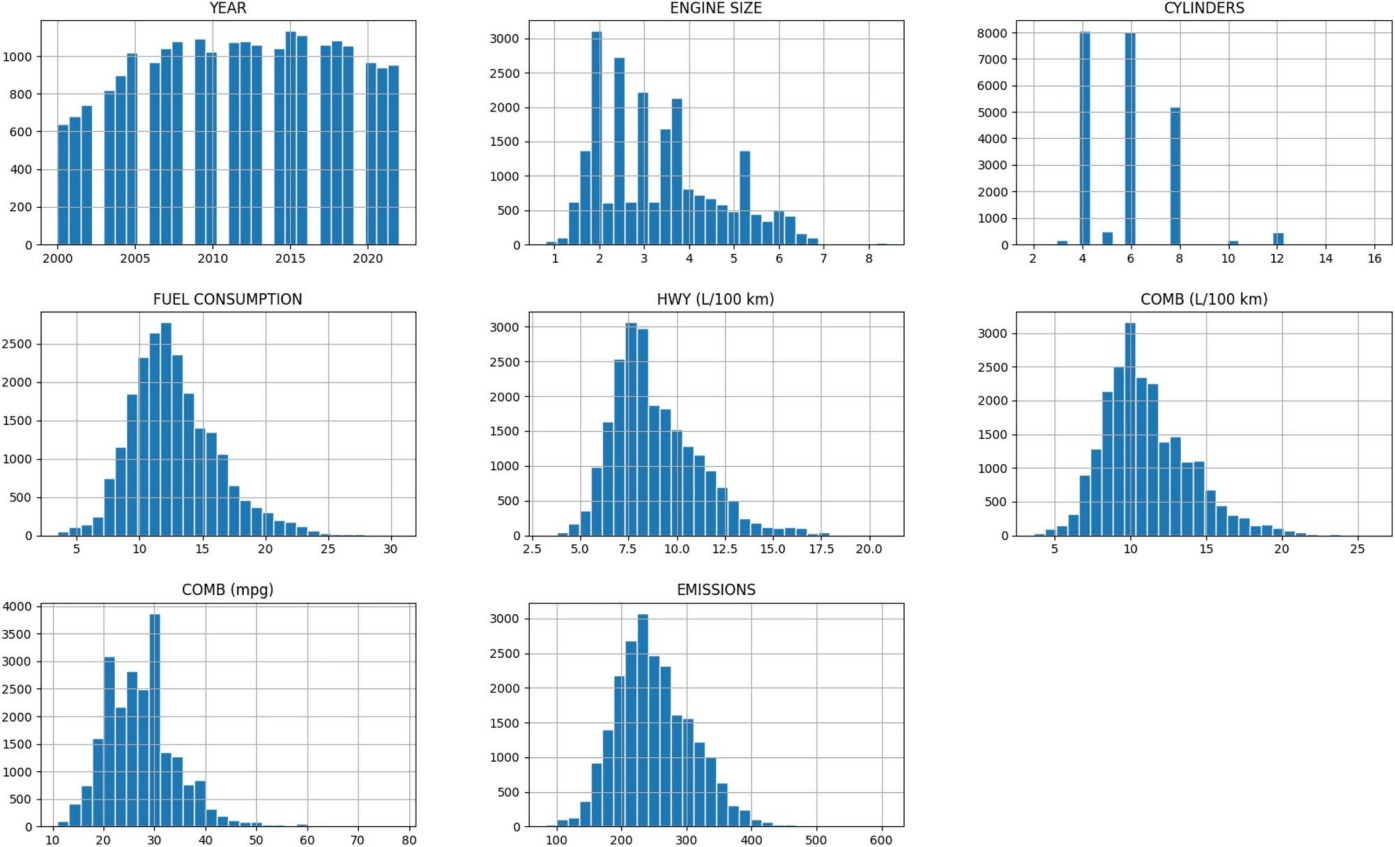
Distribution of key vehicle attributes.

Figure 4 presents descriptive statistics for engine size, number of cylinders, fuel rates (city, highway, and combined), fuel efficiency in terms of mpg, and $\hbox {CO}_2$ emission rates in the dataset. These histograms provide a skeletal framework of how the given attributes are distributed in the dataset, showing the frequency of occurrence of vehicles with certain specifications. For example, the histogram for engine size is shifted to the right, reflecting that a more significant number of vehicles are equipped with smaller engines. At the same time, the histogram for $\hbox {CO}_2$ emissions is shifted to the left, reflecting recent trends as producers improve car efficiency and decrease the negative impacts on the environment.

These exploratory results suggest an interdependence between vehicle characteristics, fuel type, and road conditions regarding fuel consumption and $\hbox {CO}_2$ emissions. Knowledge of these relationships is a foundation for developing mathematical simulations that estimate emissions over various operating modes and types of motor vehicles. Ideally, such models can be used to reveal trends that may help create environmental policies. These policies might include encouraging alternative fuel sources, improving vehicle structures, and maximizing the total environmental benefits of the transport subsector.

## Material and methods

**Fig. 5:**
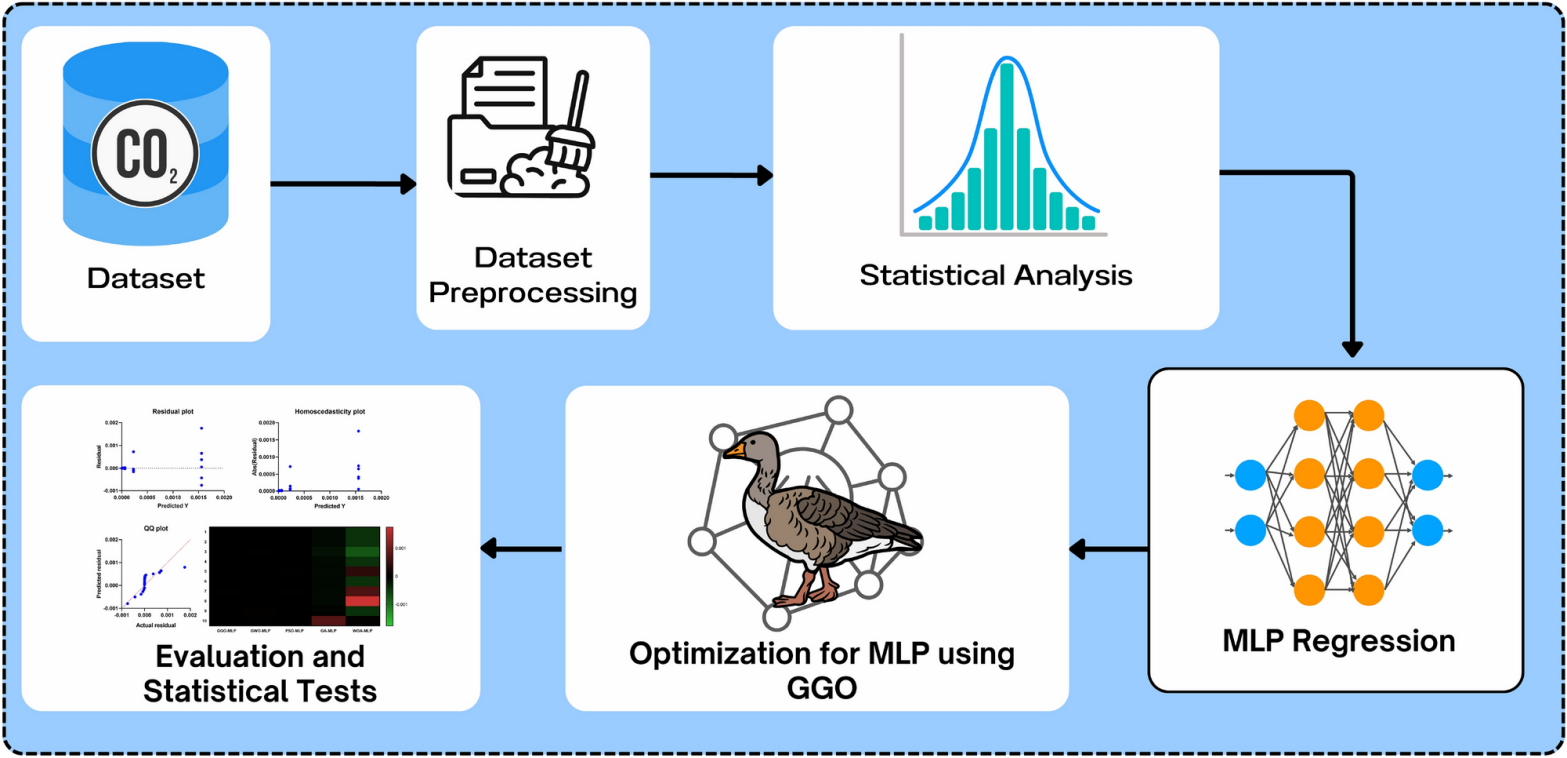
Framework of the paper.

In this section, we present the methodology employed to optimize the prediction of $\hbox {CO}_2$ emissions from Electric Vehicles (EVs) using machine learning and metaheuristic optimization algorithms. The approach begins with data collection and preprocessing, where essential vehicle characteristics are standardized to create a uniform dataset for model input. Next, we apply the Multi-Layer Perceptron (MLP) model for predicting $\hbox {CO}_2$ emissions, which is then optimized using a variety of metaheuristic algorithms, including Greylag Goose Optimization (GGO), Grey Wolf Optimization (GWO), Particle Swarm Optimization (PSO), Genetic Algorithm (GA), and Whale Optimization Algorithm (WOA). These optimization techniques are utilized to fine-tune the hyperparameters of the MLP model in order to enhance its prediction accuracy. Finally, model performance is assessed using several evaluation metrics, such as Mean Squared Error (MSE), Root Mean Squared Error (RMSE), and the coefficient of determination ($R^2$), which help identify the most effective model for $\hbox {CO}_2$ emissions prediction. The overall methodology is summarized in the framework shown in Fig. 5, which provides a visual overview of the study’s process.

### Machine learning models

Our sample analysis of detailed vehicle data for $\hbox {CO}_2$ emission prediction involved carefully analyzing five machine learning models. Out of the models we used for our experiments, the following are the models: MLPRegressor, DecisionTreeRegressor, K-Nearest Neighbors Regressor (KNN), RandomForestRegressor, and Support Vector Regressor (SVR). Each model was selected intentionally for their ability to handle non-linear relations and high-dimensional data. Within the context of these different approaches, our goal was to ascertain which of the models is the most suitable for estimating $\hbox {CO}_2$ emissions contingent on important vehicle characteristics that involve fuel type, type of transmission, size of the engine, and fuel consumption ranking^61–64^. Familiarity with how these factors play a role can enhance current emission models for the transport sector and aid in planning tactics to mitigate adverse environmental effects.

To adopt multiple criteria for the evaluation of the performance of every model, we utilized such parameters as Mean Absolute Error (MAE), Mean Squared Error (MSE), Root Mean Squared Error (RMSE), and the coefficient of determination ($R^2$). These give a complete picture of the performance of each model, including how it corrects its errors. MAE takes the arithmetic mean of the absolute errors (AE) and provides a clear and easy-to-understand measure of prediction precision. While comparing models, MSE squares the errors, emphasizing more significant discrepancies and highlighting low-quality models that do not handle significant deviations well. RMSE is derived from MSE by taking its square root, which gives a more interpretable statistic without overstressing insignificant errors. The $R^2$ statistic measures the proportion of variance in the dependent variable explained by the independent variables, providing insight into the model’s model’s explanatory power.

- *MLPRegressor (Multi-Layer Perceptron Regressor):*
  ANN, known as MLP Regressor, is mainly used to solve regression problems. It comprises an input layer, one or more hidden layers and an output layer. Only the neurons in the successive layer are connected to any neuron in a given layer. The network adjusts itself (by adjusting the weights of these connections), minimizing prediction errors in a process called backpropagation, which is based on a gradient descent algorithm.
  The MLPRegressor can estimate $\hbox {CO}_{{2}}$ emissions with coexisting and interrelated dependencies in data by capturing nonlinear couplings within data, for instance, fuel type, engine size and transmission type. The models were extended in the current study by varying the number of hidden layers and neurons per layer and/or using activation functions other than ReLU (Rectified Linear Unit), such as the tanh. We also learned to change the learning rate at which weight updates act during each learning step to make the convergence happen. We employed techniques like L2 regularization to counteract overfitting, which helps reduce the network weights so that the model is more straightforward and performs better when testing.
  During the MLP training process, backpropagation techniques are used to correct the errors committed in a prediction and to give it a better result in future forecasting. This enables the MLPRegressor to learn and replicate the data details that a simpler model cannot learn or predict. Moreover, MLP provides flexibility to model complex regression problems (such as $\hbox {CO}_2$ emissions prediction) using multiple architectures and training algorithms.
  I chose MLPs among other neural network structures because MLP structures have the right balance of complexity and efficiency. Since MLP is not specialized for image or sequential data, unlike more specialized architectures like Convolutional Neural Networks (CNNs) or Recurrent Neural Networks (RNNs), it’s an excellent fit for tabular datasets, such as the one in this study. The MLP is well suited to deal with the highly nonlinear and dependent relationships among features such as fuel type, vehicle specifications, and driving conditions in the $\hbox {CO}_2$ emissions data.
  MLPRegressor is also lighter on computational resources for large-scale experiments involving hyperparameter optimization than deeper architectures. In addition to being easily trainable to fit complex regression tasks, it can also accommodate different hidden layer configurations and activation functions without the overhead of more complex networks. Because of this, the MLP model is the best and most efficient choice for this study, a substantial tradeoff between accuracy and computational feasibility.
- *Decision Tree Regressor:*
  DecisionTreeRegressor is a straightforward but powerful algorithm that builds a tree structure feature by feature by partitioning the data, minimizing the measure of deviations from the target variable at each node. It recursively chops the data into partitions and builds a tree of decisions explaining the predicted variable at the leaves of the tree.
  The one big plus of the Decision Tree Regressor is interpretation. It allows visualization of the tree, showing the decision made at an individual node. This feature allows $\hbox {CO}_2$ emissions prediction, providing helpful information on which vehicle characteristics are the most determining. However, decision trees can be deep and have no constraints to bind the depth they can get. To compensate for this, we tweaked parameters like the maximum depth of the tree, the minimum number of cases to create a terminal node, and the minimum number of cases to split a node. With these adjustments, the tree was still generalized but could identify patterns in that dataset.
- *K-Nearest Neighbors (KNN):*
  The KNN algorithm is a simple, non-parametric technique where predictions are based on the *k* closest data points in the feature space. It measures the ‘distance’ between the test data and the dataset using standard distance metrics (e.g., Euclidean distance: $\sqrt{(x_2 - x_1)^2 + (y_2 - y_1)^2}$), selects the *k* nearest neighbors, and computes the mean of their target values.
  KNN is straightforward to implement, making it a suitable baseline for regression problems, including $\hbox {CO}_2$ emission prediction. It uses attributes such as fuel consumption, power, number of cylinders, body type, fuel type, and transmission type to estimate $\hbox {CO}_2$ outputs. While KNN is simple, it is computationally expensive for large datasets as distances need to be calculated for all predictions. We optimized the value of *k* to balance bias and variance, ensuring the model was neither too noisy (small *k*) nor too smooth (large *k*). Additionally, we evaluated the effect of distance metrics like Manhattan and Minkowski on system accuracy.
- *RandomForestRegressor:*
  The RandomForestRegressor combines multiple decision trees and calculates the mean output of all the trees for robust predictions. Each tree is trained on a random subset of the data, and random subsets of features are considered for splitting nodes. This randomness prevents all trees from focusing on the same features, reducing overfitting and improving generalization.
  RandomForestRegressor is effective for data with complex nonlinear relationships, making it suitable for $\hbox {CO}_2$ emission prediction. Combining predictions from multiple trees reduces the likelihood of significant errors and improves reliability. In our study, we optimized parameters like the number of trees in the forest, maximum tree depth, and the minimum number of samples required for splitting. Additionally, feature importance analysis within the model revealed which factors contributed most to $\hbox {CO}_2$ emissions, aiding policy development and engineering control.
- *SVR (Support Vector Regressor):*
  Support Vector Machines (SVM) have been previously discussed; SVR stands for Support Vector Regression, which applies SVM principles to regression analysis. Its primary intent is to identify a hyperplane that maximizes the distance from the data points with the highest density. SVR incorporates a controlled error bound ($\epsilon$) to minimize noise and improve the distinctions between predictions and outcomes.
  It has been observed that SVR works fine with very high-dimensional and complicated datasets, often using some commonly available kernel functions like the Radial Basis Function (RBF). In particular, the RBF kernel is suitable for encoding the nonlinear features in the dataset due to its capability to predict the $\hbox {CO}_2$ emission levels. Additionally, we tuned parameters such as *C*, which defines how much we want to minimize error or $\epsilon$, which is the margin of tolerance around the hyperplane. Detailed regression tasks are best suited to SVR because they work well with complex datasets, and the risk of overfitting is low.

Each of the five machine learning models was selected because they cover different elements and approaches to predicting $\hbox {CO}_2$ emissions. Of these models, MLPRegressor brought a winning streak for nonlinear models, while DecisionTreeRegressor was easy to interpret, KNN was easy to use and required a lot of computational power for large datasets, RandomForestRegressor added robustness and usefulness of feature importance analysis, and last but not least the SVR was accurate and capable to model nonlinearly in predicting $\hbox {CO}_2$ emissions. Taken together, this analysis paves the way for the application of machine learning in environmental modeling and helps make policies and engineering principles more sustainable.

#### Evaluation metrics

To evaluate the extent of how well each machine learning model predicts $\hbox {CO}_2$ emissions, we employed a complete set of evaluation metrics. The detailed metrics provide high-level insight into model performance, particularly for model prediction reliability, error dispersion, and model execution efficiencies. The evaluation measures used are Mean Squared Error (MSE), Root Mean Squared Error (RMSE), Mean Absolute Error (MAE), Pearson correlation coefficient (*r*), coefficient of determination ($R^2$), Relative Root Mean Squared Error (RRMSE), Nash-Sutcliffe Efficiency (NSE), Willmott Index of Agreement (WI) and Fit Time (s). Evaluating all metrics highlights different features of model performance and represents a complete basis for choosing the model with the highest predictive performance of $\hbox {CO}_2$ emissions.

1. *Mean Squared Error (MSE)*
  *Definition and Justification:* MSE measures the average squared differences between predicted and actual values:
  $$\begin{aligned} \text {MSE} = \frac{1}{n} \sum _{i=1}^{n} (y_i - \hat{y}_i)^2 \end{aligned}$$
  MSE was chosen for its ability to highlight significant errors, ensuring that large deviations in predictions are minimized. This is crucial in applications such as $\hbox {CO}_2$ emissions forecasting, where substantial errors could lead to misleading conclusions.
2. *Root Mean Squared Error (RMSE)*
  *Definition and Justification:* RMSE is the square root of MSE, providing an error measure in the same units as the target variable:
  $$\begin{aligned} \text {RMSE} = \sqrt{\text {MSE}} \end{aligned}$$
  RMSE was selected for its interpretability and ability to reflect the overall error magnitude, which helps to compare model performances tangibly.
3. *Mean Absolute Error (MAE)*
  *Definition and Justification:* MAE calculates the average magnitude of prediction errors without considering their direction:
  $$\begin{aligned} \text {MAE} = \frac{1}{n} \sum _{i=1}^{n} \left| y_i - \hat{y}_i \right| \end{aligned}$$
  MAE was included because it is simple and focuses on the average prediction error, providing an intuitive measure of model reliability.
4. *Pearson Correlation Coefficient (r)*
  *Definition and Justification:* The *r* coefficient measures the strength and direction of the linear relationship between predicted and actual values. Values close to 1 or -1 indicate strong linear relationships, while values near 0 indicate weak relationships. Including *r* allows us to assess how well the model captures the trends in the data.
5. *Coefficient of Determination (*$R^2$*)*
  *Definition and Justification:* $R^2$ measures the proportion of variability in the target variable explained by the model:
  $$\begin{aligned} R^2 = 1 - \frac{\sum _{i=1}^{n} (y_i - \hat{y}_i)^2}{\sum _{i=1}^{n} (y_i - \bar{y})^2} \end{aligned}$$
  $R^2$ was chosen for its ability to evaluate the overall fit of the model and its explanatory power.
6. *Relative Root Mean Squared Error (RRMSE)*
  *Definition and Justification:* RRMSE normalizes RMSE by the mean of actual values, providing a relative error measure. This metric ensures that errors are comparable across datasets with different scales.
7. *Nash-Sutcliffe Efficiency (NSE)*
  *Definition and Justification:* NSE evaluates the predictive power of the model compared to the mean of the observed data:
  $$\begin{aligned} \text {NSE} = 1 - \frac{\sum _{i=1}^{n} (y_i - \hat{y}_i)^2}{\sum _{i=1}^{n} (y_i - \bar{y})^2} \end{aligned}$$
  NSE was included to quantify how well the model predictions match observed data, with values closer to 1 indicating better performance.
8. *Willmott Index of Agreement (WI)*
  *Definition and Justification:* WI measures the degree of agreement between predicted and observed values, taking values between 0 and 1. Higher WI values indicate better alignment between predictions and actual data, making it a valuable metric for assessing model accuracy.
9. *Fit Time (s)*
  *Definition and Justification:* Fit Time represents the computational time required to train the model. This metric was included to evaluate the computational efficiency of the models, which is crucial for practical applications requiring fast and scalable solutions.

*Choice of Hyperparameters:* Both models’ hyperparameters were selected to yield a trade-off between model complexity and generalization. For example, the number of hidden layers and neurons in the MLP model varied to find the expression mechanism of the hidden nonlinear dependencies in the data. Regularization techniques such as L2 Regression were used to prevent overfitting, and fine-tuning of the learning rate was done to meet convergence without sacrificing efficiency. We further refined these hyperparameters using the GGO optimization algorithm, which systematically searched the space for the best configuration for minimizing error metrics.

By subjecting a complete set of metrics to this process and systematically optimizing hyperparameters, we compared the strengths and weaknesses of each model. This approach to prediction modeling is multidimensional so that the most reliable and robust model can be selected for different prediction problems. This analysis is critical because accurate $\hbox {CO}_2$ emissions forecasts are critical for effective action on environmental policy decisions and sustainable transportation strategies.

### Greylag Goose Optimization (GGO)

The Greylag Goose Optimization (GGO) is a metaheuristic optimization technique based on the life, particularly the behavior, of Greylag geese. As the model mimics how the geese communicate, fly in formation, and think collectively, GGO is expected to optimally solve problems in large search spaces. It balances exploration and exploitation, thus improving its solution-finding capabilities in all optimization problems^65^.

#### Biological inspiration

The implementation of the GGO algorithm is inspired by the fundamental behavior and learning paradigms of Greylag geese (*Anser anser*). These geese are some of the most friendly, loyal, and tenacious birds, vital for roaming and migrating. Key biological behaviors influencing the GGO algorithm include:

- *Social Structure and Loyalty:* Long-term pair bonds are formed when breeding Greylag geese; these birds are constantly conjoined in groups. This provides a structural framework in which the energies and actions of individuals in the society are well harmonized to realize shared goals achieved collectively.
- *Migration Patterns:* During migration, the birds fly in a V formation, which reduces drag and improves aerodynamic efficiency. This formation allows the head of the flock and other members, typically involved in long-distance travel, to pass vital information effectively.
- *Adaptive Intelligence:* These geese demonstrate remarkable cognitive abilities, such as identifying and remembering specific signs for years. This capability enables them to adapt their pathways according to changing environmental conditions.
- *Exploration and Exploitation Balance:* The geese exhibit behaviors related to habitat selection, balancing the tendency to explore new directions for optimal conditions with the stability required to care for their young and maintain their nests.

Based on these behaviors, the GGO algorithm extracts techniques that generalize and replicate superior search behaviors, enabling the algorithm to execute sophisticated search processes for optimization problems.

### Algorithm overview

The GGO algorithm follows a structured flowchart derived from cycles consisting of five iterative steps that emulate the behaviors of Greylag geese and their interactions. These steps include identification, evaluation, categorization and allocation, decision-making and escalation, and termination. The detailed steps are outlined as follows:

1. *Initialization:*
  Create a set of individual solutions randomly selected from the initial search range. Each individual represents a potential solution to the optimization problem.
  Define algorithm-dependent parameters, including the population size (*n*), the maximum number of iterations ($t_{\text {max}}$), and other factors that determine the trade-off between exploration and exploitation.
2. *Evaluation:*
  Evaluate each individual using a predefined objective function $F_n$ to determine the quality of the optimization problem.
  Select the most promising solution among the current population, called the *leader*, denoted as *F*(*P*), for use in the subsequent iteration.
3. *Group Allocation:*
  Dynamically assign individuals to two distinct groups: $n_1$ for exploration and $n_2$ for exploitation, such that $n = n_1 + n_2$.
  The initial budget split is typically 50/50 (exploration/exploitation), but this may adjust as the algorithm progresses.
4. *Exploration and Exploitation Phases:*
  *Exploration Phase:* Focuses on finding better areas to explore for improved solutions, thus avoiding local optima.
  *Exploitation Phase:* Concentrates on refining current best solutions to make incremental enhancements.
5. *Iteration:*
  Repeat the assessment and improvement cycle for a fixed number of iterations or until convergence criteria are met, such as no significant improvement in the leader fitness over a specified number of cycles.
6. *Termination:*
  The algorithm terminates when the maximum number of iterations is reached or when convergence criteria are satisfied.
  Return the best-found solution as the optimal solution to the problem.

#### Exploration operation

The exploration phase is crucial for broadening the search scope and ensuring the algorithm is not trapped in local optima. Inspired by the geese’s exploratory behavior when seeking new favorable spots, GGO employs several strategies to enhance exploration:

*Position Update Mechanism*    The following equation governs the primary update rule for exploration:

1 $$\begin{aligned} X(t+1) = X^*(t) - A \cdot |C \cdot X^*(t) - X(t)| \end{aligned}$$

where

- *X*(*t*): Current position of the agent at iteration *t*.
- $X^*(t)$: Position of the leader (best solution) at iteration *t*.
- $A = 2a \cdot r_1 - a$: Control parameter influencing the exploration magnitude.
- $C = 2 \cdot r_2$: Scaling factor for the position update.
- $r_1, r_2$: Random values uniformly distributed in the interval [0, 1].
- *a*: Parameter linearly decreases from 2 to 0 over iterations, controlling the balance between exploration and exploitation.

This mechanism ensures that agents are attracted toward the leader while maintaining a stochastic component to encourage diverse exploration.

*Enhanced Exploration Through Multi-Agent Interaction*    To further augment the exploration capability, GGO incorporates interactions among three randomly selected search agents, referred to as “Paddles.” The update rule in this context is given by:

2 $$\begin{aligned} \begin{aligned} X(t + 1)&= w_1 X_{\text {Paddle1}} + z w_2 (X_{\text {Paddle2}} - X_{\text {Paddle3}}) \\&+ (1 - z) w_3 (X - X_{\text {Paddle1}}) \end{aligned} \end{aligned}$$

where

- $w_1, w_2, w_3$: Weight parameters dynamically updating within the range [0, 2], controlling the influence of each paddle.
- $z = 1 - \left( \frac{t}{t_{\text {max}}}\right) ^2$: Exponentially decreasing factor that modulates the influence of different terms as iterations progress.
- $X_{\text {Paddle1}}, X_{\text {Paddle2}}, X_{\text {Paddle3}}$: Positions of three randomly chosen agents from the population, facilitating diverse information exchange.

*Stochastic Diversification*    To introduce randomness and prevent premature convergence, the position update incorporates stochastic elements when a certain condition is met ($r_3 \ge 0.5$):

3 $$\begin{aligned} \begin{aligned} X(t + 1)&= w_4 | X^*(t) - X(t) | e^{bl} \cos (2\pi l) + 2 w_1 (r_4 + r_5) X^*(t) \end{aligned} \end{aligned}$$

where

- $w_4$: Weight parameter within [0, 2].
- *b*: Constant influencing the exponential term.
- $l \in [-1, 1]$: Random value introducing oscillatory behavior through the cosine function.
- $r_4, r_5$: Random values within [0, 1] contributing to the stochastic component.

This stochastic diversification ensures that the algorithm maintains a balance between exploration and exploitation, especially in the later stages of the search process.

#### Exploitation operation

The exploitation phase focuses on intensifying the search for the best solutions to fine-tune and enhance their quality. This phase leverages the collective intelligence of the flock to converge toward optimal solutions through two primary strategies:

*Moving Towards the Best Solution*    In this strategy, individuals in the exploitation group are guided toward the leader’s position by leveraging the positions of “sentry” agents. The updated rules are as follows:

4 $$\begin{aligned} \left\{ \begin{aligned} X_1&= X_{\text {Sentry1}} - A_1 \cdot |C_1 \cdot X_{\text {Sentry1}} - X| \\ X_2&= X_{\text {Sentry2}} - A_2 \cdot |C_2 \cdot X_{\text {Sentry2}} - X| \\ X_3&= X_{\text {Sentry3}} - A_3 \cdot |C_3 \cdot X_{\text {Sentry3}} - X| \\ \end{aligned} \right. \end{aligned}$$

where

- $X_{\text {Sentry1}}, X_{\text {Sentry2}}, X_{\text {Sentry3}}$: Positions of three designated sentry agents within the exploitation group.
- $A_1, A_2, A_3 = 2a \cdot r_1 - a$: Control parameters for each sentry agent.
- $C_1, C_2, C_3 = 2 \cdot r_2$: Scaling factors for each sentry agent.

The updated position for each agent in the exploitation group is then calculated as the average of these three updated positions:

5 $$\begin{aligned} X(t+1) = \frac{1}{3} \sum _{i=1}^{3} X_i \end{aligned}$$

This averaging process ensures that the exploitation group converges towards the most promising regions identified by the sentry agents.

*Searching the Area Around the Best Solution*    This strategy involves a localized search around the leader’s vicinity to fine-tune the solution:

6 $$\begin{aligned} X(t + 1) = X(t) + D(1 + z) w (X - X_{\text {Flock1}}) \end{aligned}$$

where

- *D*: Scaling factor adjusting the magnitude of the positional update.
- $z = 1 - \left( \frac{t}{t_{\text {max}}}\right) ^2$: Exponentially decreasing factor reducing the influence of this term over iterations.
- *w*: Weight parameter controlling the extent of the local search.
- $X_{\text {Flock1}}$: Position of a focal agent or a subset of the flock guiding the local search.

This approach guarantees a much tighter function for exploring the neighborhood around the best solutions, augmenting the optimization algorithm’s capacity to tweak and arrive at the optimum.

#### Dynamic group allocation and adaptation

The last but not the least essential distinctive feature of GGO is a continuous and random shift of employees from the exploration to the exploitation subset. According to the algorithm’s progress, this allocation adjusts to ensure that an adequate number of solutions are found while simultaneously improving upon existing solutions. The adaptation mechanism includes:

- *Initial Allocation*: May also be initiated with an equal level of exploration (e.g., 50 percent and 50 percent exploitation).
- *Adaptive Adjustment*: If the leader’s fitness remains unchanged for a predefined number of consecutive iterations (e.g., three iterations), the algorithm increases the size of the exploration group ($n_1$) to encourage the discovery of alternative solutions, preventing stagnation and limiting cycles.
- *Random Reassignment*: Sometimes, agents may interchange the roles of the differing groups according to raffle assignments to eliminate quick stabilization.

Such dynamic adaptation is particularly effective since it improves GGO’s value in working with complex and dynamic search environments.

#### Algorithm pseudo-code

The operational flow of the GGO algorithm is encapsulated in Algorithm 1. The initialization, evaluation and group allocation phase, the exploration and exploitation updates phase, and the termination phase of this detailed representation incorporate the following procedures.

**Algorithm 1:**
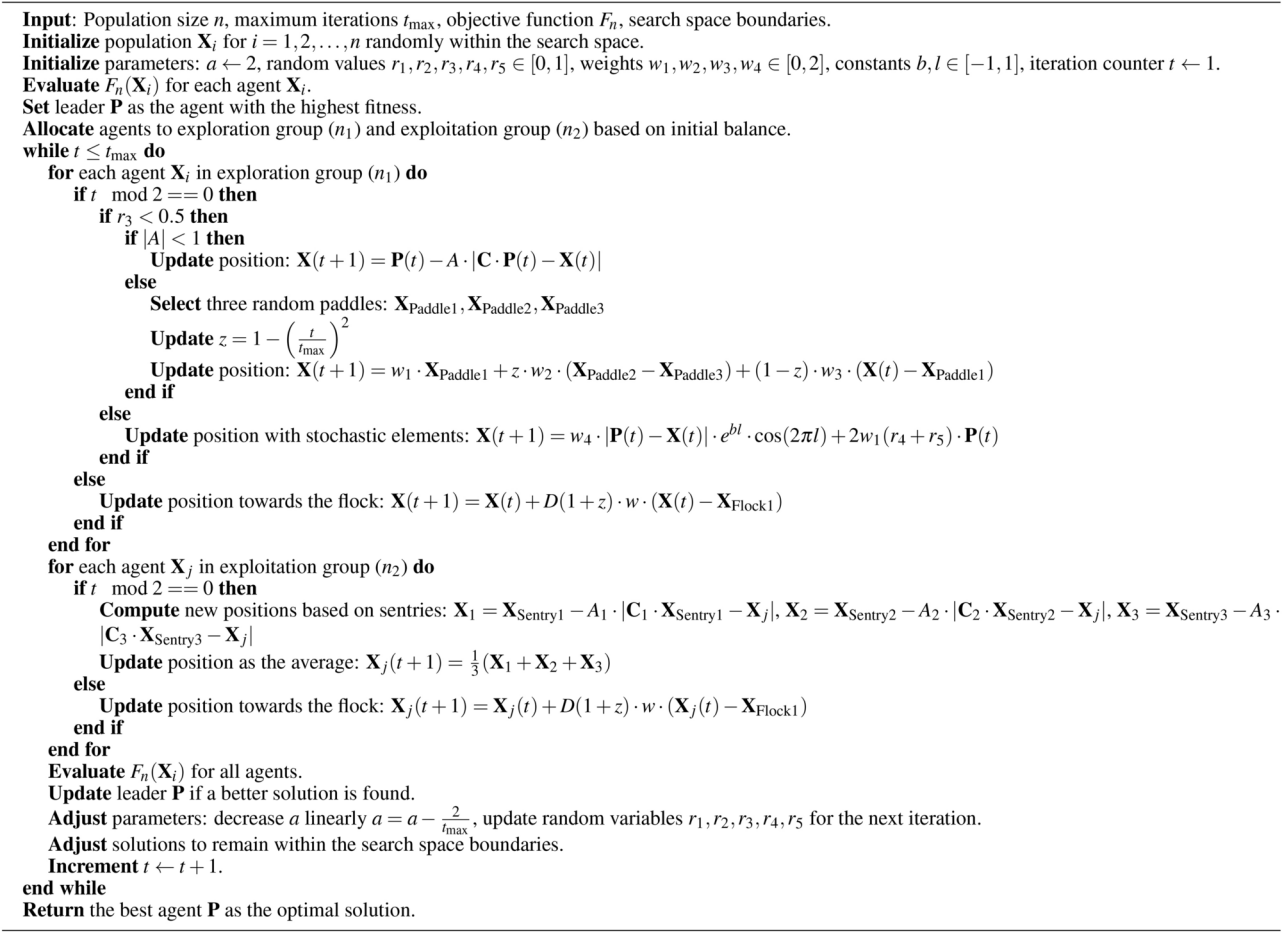
Greylag Goose Optimization (GGO) Algorithm

#### Complexity analysis

Familiarity with the computational complexity of the GGO algorithm forms a major objective of this evaluation, particularly when applied to large-scale optimization problems. The complexity analysis is as follows:

- *Initialization*:
  - Also, the initial population of *n* agents in the process of genetic algorithm initialization implies the random placement of individuals in the search space. If *O*(1) initialization for each agent, the initialization time complexity is *O*(*n*).
- *Objective Function Calculation*:
  - Evaluating the objective function $F_n$ for each of the *n* agents incurs a complexity of *O*(*n*) per iteration.
- *Exploration and Exploitation Updates*:
  - Updating the positions of agents in both exploration ($n_1$) and exploitation ($n_2$) groups involves a constant number of operations per agent, leading to a complexity of *O*(*n*) per iteration. Such conditional checks and stochastic updates do not even amount to a constant factor in the total computational complexity.
- *Parameter Updates and Leader Selection*:
  - Updating parameters such as $a, r_i$, weight factors, and selecting the new leader, each contributes *O*(*n*) operations per iteration.
- *Total Complexity*:
  - The objective function evaluatons are *O*(*n*) operations and updating positions and parameters *O*(*n*) each offering a total *O*(*n*) per iteration.
  - With $t_{\text {max}}$ iterations, the overall computational complexity of the GGO algorithm is $O(t_{\text {max}} \cdot n)$.

The linear computational complexity regarding population size and iteration number also indicates that GGO suits a broad range of optimization problems.

The Greylag Goose Optimization (GGO) algorithm addresses the natural question of how social and adaptive behavioral patterns in Greylag geese can be utilized to solve complex problems. Understanding its mechanical model makes its approach easier to appreciate. GGO achieves near-optimal and robust performance in different optimization problems due to the MOEA paradigm, incorporating exploration and exploitation, multi-agent interactions, and stochastic diversification mechanisms. This makes it useful in research and practice because of its flexibility, simplicity, and applicability to large-scale data. There is significant promise in the current and new changes in hybridization to produce better and more optimal performance, making the GGO more stable within the family of accurate metaheuristic optimization algorithms.

### Potential of hybridization of GGO

Due to its attributes facilitating a balance between exploration and exploitation, the Greylag Goose Optimization (GGO) algorithm has great potential to be hybridized with other optimization algorithms to yield improved performance. Hybridization combines the strengths of several algorithms, taking advantage of their complementary characteristics to compensate for the shortcomings of single-component algorithms. Below are some approaches to hybridize GGO with other algorithms:

#### GGO-PSO hybrid

The hybrid strategy mixes GGO and PSO. In this strategy, additional exploration is used to seek a local optimum near the global optimum found by GGO, thereby improving GGO’s global search capabilities.

Particle Swarm Optimization (PSO) is widely known for its fast convergence but frequently suffers from premature convergence in complex, multimodal problems. By combining GGO’s adaptive exploration capabilities with PSO’s velocity update mechanism, a new GGO-PSO hybrid is introduced, where GGO could dynamically control the exploration/exploitation balance. For example:

- Initialize the population using GGO and then use it to guide an exploration phase to find promising regions in the search space.
- Utilize PSO’s particle velocity and position updates during the exploitation phase to fine-tune the solutions where convergence is faster.

This hybridization could be highly effective for problems where search space diversity is a key aspect.

#### GGO-GA hybrid

Genetic Algorithms (GA) maintain diversity through genetic operations like crossover and mutation but are computationally expensive. By combining GGO’s structured exploration mechanisms with GA’s genetic diversity enhancement, the GGO-GA hybrid offers promising results. For example:

- Use GGO for the initial exploration to locate clusters of potential solutions.
- Apply GA’s crossover and mutation operators to these clusters to inject variability and refine the solutions.

This approach improves robustness by combining GGO’s global search efficiency with GA’s ability to escape local optima.

#### GGO-ML algorithms hybrid

A hybrid approach combining GGO and Machine Learning (ML) algorithms can enhance optimization, particularly in hyperparameter tuning. GGO can optimize parameters to boost the performance of ML algorithms like Neural Networks and Gradient Boosting. For example:

- Use GGO to automatically tune parameters such as learning rate, number of estimators, and hidden layer dimensions.
- Incorporate these optimized parameters into ML models to enhance generalization and predictive accuracy.

This hybridization improves the performance of ML models for accurate prediction in applications such as $\hbox {CO}_2$ emissions forecasting.

#### Dynamic hybrid models

Adaptive selection in dynamic hybrid models can switch between GGO and other algorithms based on convergence criteria or problem characteristics. For example:

- Start with GGO to explore the search space and discover promising regions.
- Transition to WOA (Whale Optimization Algorithm) or GWO (Grey Wolf Optimizer) in later stages for exploitation to fine-tune the solutions.

This dynamic switching ensures that the hybrid algorithm can benefit from the strengths of diverse techniques and overcome their shortcomings.

### Advantages of hybridization

By hybridizing GGO with other algorithms, the following advantages can be achieved:

- Complementary algorithmic mechanisms to further accelerate convergence speed.
- Enhanced solution quality by exploiting the strengths of multiple optimization paradigms.
- Improved robustness and adaptability for solving complex, dynamic, and multimodal optimization problems.

*Future Directions*    Future work could explore the development of hybrid GGO methods for various applications, such as multi-objective optimization, high-dimensional problems, and time-sensitive computations. Hybridization with ensemble methods and adaptive mechanisms is also expected to yield further performance gains.

GGO can be easily integrated with other algorithms, increasing its potential for achieving superior optimization performance. Thus, it becomes a versatile tool for solving various complex real-world problems.

## Results

This section presents the accuracy of the current and optimized machine learning models for $\hbox {CO}_2$ emissions. We began by evaluating five fundamental regression models: the tested regression models included MLPRegressor, DecisionTreeRegressor, K-Nearest Neighbors, RandomForestRegressor, and Support Vector Regressor (SVR)^41–43,66^. These were evaluated using standard performance statistics, including Mean Squared Error (MSE), Root Mean Squared Error (RMSE), Mean Absolute Error (MAE), correlation coefficient (*r*), coefficient of determination ($R^2$), Relative Root Mean Squared Error (RRMSE), Nash Sutcliffe Efficiency (NSE), and Willmott’s Index of Agreement (WI).

After the initial assessment, we examined how selecting the best optimization algorithm for MLP-GGO, GWO, PSO, GA, and WOA-improved its reliability^61–64^. We used the same performance measures to compare the optimized models and then performed t-tests to evaluate the significance level between the models. To ensure computational efficiency and reproducibility, all experiments were conducted on Google Colab computational services using the NVIDIA A100 GPU, leveraging its high-performance computing capabilities for accelerated model training and optimization.

To ensure a fair and consistent comparison among the various optimization algorithms utilized in this study, it is crucial to establish standardized initial parameter values and ranges. The selection of these parameters significantly influences the performance and convergence behavior of the optimization techniques. The population size, number of iterations, and number of independent runs have been uniformly set across all algorithms to maintain experimental consistency. Additionally, algorithm-specific control parameters have been initialized based on literature recommendations and prior experimental findings to optimize exploration and exploitation capabilities.

Table 3 provides a comprehensive overview of the initial parameter values and their respective ranges for each optimization algorithm considered in this study. These parameters govern the dynamic behavior of each metaheuristic approach, ensuring optimal performance in tuning machine learning models for $\hbox {CO}_2$ emissions prediction. The table includes details on control factors such as weight coefficients, random variables, exploration-exploitation balance, and mutation probabilities, which play a critical role in guiding the search process toward optimal solutions.

**Table 3:** Initial parameter values and ranges for various optimization algorithms.

Algorithm	Parameter	Value/range
All algorithms	Population size	30
	Number of iterations	500
	Number of runs	30
GGO	Control parameter (*a*)	Linearly decreasing from 2 to 0
	Random values ($r_1, r_2$)	[0,1]
	Weight parameters ($w_1, w_2, w_3$)	[0,2]
	Weight parameter ($w_4$)	[0,2]
	Random values ($r_4, r_5$)	[0,1]
	Random variable (*l*)	$[-1,1]$
	Random parameter ($r_3$)	[0,1]
BER	Size of population	30
	Iterations count	500
	Mutation probability	0.5
	Exploration percentage	70
	*K* (decreases from 2 to 0)	1
	Number of runs	30
GWO	*a*	2 to 0
WWPA	Evaporation rate ($\lambda$)	[0.1, 0.5]
	Water flow rate ($\phi$)	[0.1, 0.9]
	Maximum capacity ($C_{\max }$)	[1, 5]
HHO	Energy parameter (*E*)	$[-1, 1]$
	Escape energy ($E_0$)	$[-1, 1]$
	Levy flight coefficient ($\beta$)	1.5
PSO	Inertia ($W_{\text {max}}, W_{\text {min}}$)	[0.9, 0.6]
	Acceleration constants ($C_1, C_2$)	[2, 2]
JAYA	Variable range ($x_i$)	$[-100,100]$
	Random numbers ($r_1, r_2$)	[0,1]
DTO	Step size ($\delta$)	[0.01, 0.1]
	Exploration factor ($\alpha$)	[0.5, 1]
	Adaptive parameter ($\eta$)	[0.1, 0.9]
GA	Mutation probability	0.05
	Crossover rate	0.02
SFS	Fractal dimension ($D_f$)	[1.2, 1.8]
WOA	*a*	2 to 0

Table 4 reports the baseline regression models’ accuracy statistics in predicting $\hbox {CO}_2$ emissions. These metrics provide information about how accurate and reliable each model is and how fast they can perform the computations.

**Table 4:** Performance metrics for baseline regression models in predicting $\hbox {CO}_2$ emissions.

Model	MSE	RMSE	MAE	*r*	$R^2$	RRMSE	NSE	WI	Fit time (s)
MLP	0.003632	0.019058	0.014749	0.97836	0.976861	1.019416	0.956861	0.904336	5.033147
Decision Tree	0.005338	0.023104	0.017933	0.972849	0.965992	1.023539	0.945992	0.903995	7.726721
KNN	0.006687	0.025859	0.018748	0.968468	0.957399	2.026345	0.941740	0.905831	9.407264
Random Forest	0.016962	0.041185	0.034009	0.964425	0.891939	3.041959	0.931939	0.772682	10.401910
SVR	0.030546	0.055268	0.050311	0.957443	0.805403	4.056307	0.928054	0.667838	12.144048

From the results, the MLPRegressor computes with the least MSE of 0.0036 and RMSE of 0.0191 and is deemed the most appropriate model for determining $\hbox {CO}_2$ emissions. This suggests high accuracy compared to the other models under investigation in the study. The high values of *r*, also supported by a confirmed $R^2$, indicate the high capability of the MLPRegressor to approximate the examined relationship between the predicted and actual values. An $R^2$ value near 1 indicates that the model has captured a high percentage of the variability in the data. Furthermore, the moderate computational time taken by the MLPRegressor, approximately 5 seconds, makes it suitable when both accuracy and computational speed are significant.

The DecisionTreeRegressor and KNN models also performed exceptionally well, although they had lower $R^2$, MSE, and RMSE scores than the MLPRegressor. These models retained a relatively high measure of goodness-of-fit as measured by the correlation coefficients, suggesting they can capture essential data features, though not with equal precision. The DecisionTreeRegressor deserves special attention for its interpretability in terms of features and decision-making paths. However, while KNN is easy to understand and implement, its accuracy can be unstable for different values of *k* and varying data distributions.

On the other hand, both the RandomForestRegressor and SVR exhibited higher error rates and lower correlation coefficients, indicating these algorithms might be less effective for this particular dataset. The RandomForestRegressor, which models dependencies well and can capture higher-degree polynomials, showed higher prediction errors, likely due to suboptimal hyperparameters or data characteristics. Meanwhile, despite its advantages, the proposed SVR demonstrated higher error rates and longer computational times than the other methods, making it less suitable for time-critical applications.

**Fig. 6:**
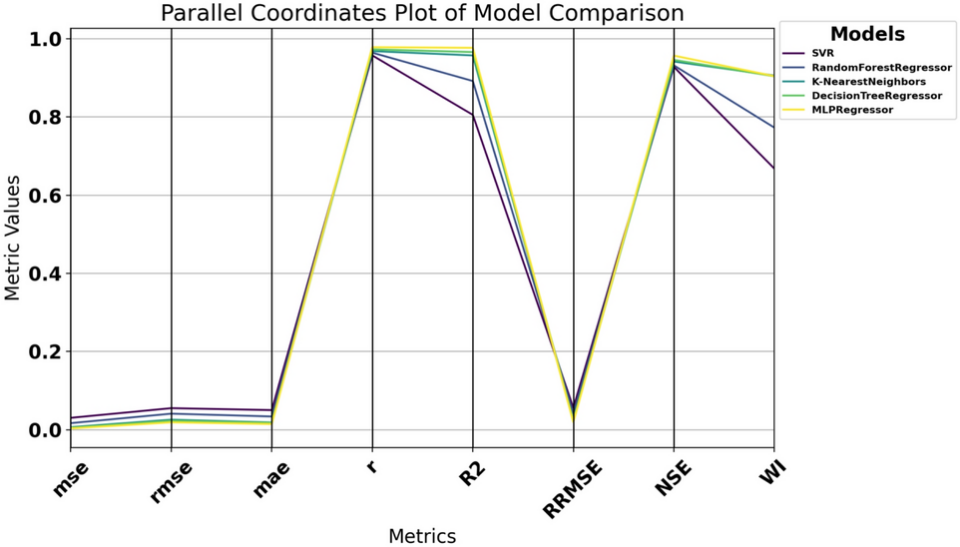
Parallel coordinates plot comparing baseline regression models.

Figure 6 provides a comparative visualization of the performance of the baseline models based on the discussed metrics. It highlights the superior accuracy and computational efficiency of the MLPRegressor relative to the other models.

**Fig. 7:**
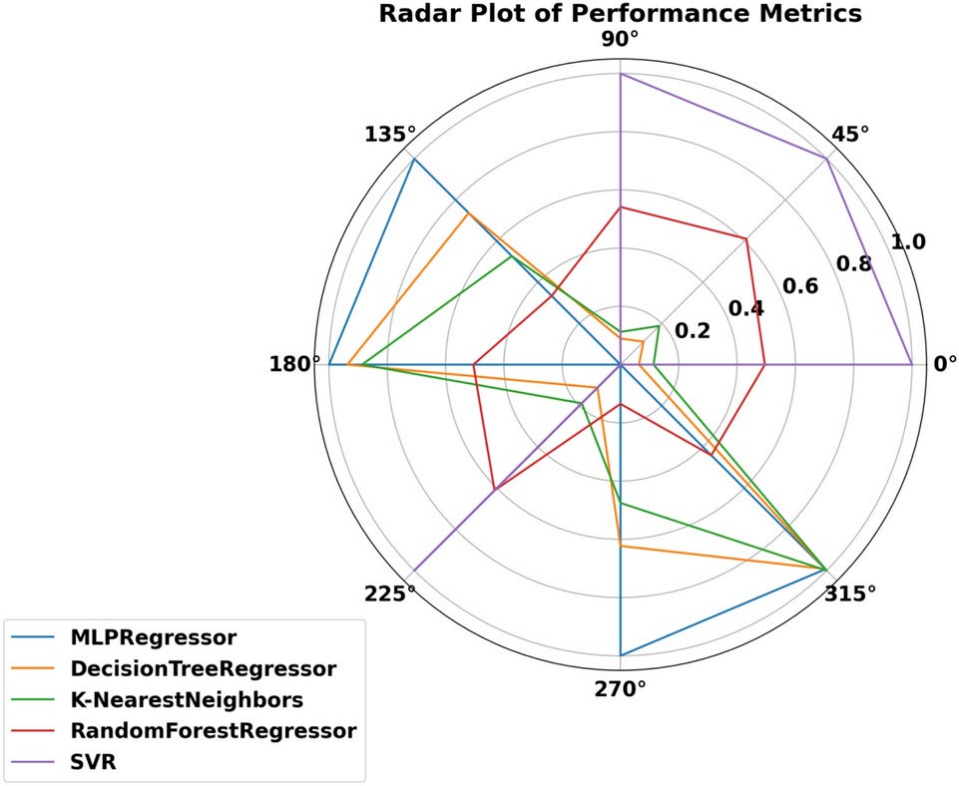
Radar plot of performance metrics for baseline models.

The radar plot in Fig. 7 summarizes each model’s performance across the critical evaluation metrics. Each axis represents a different metric, and the plot highlights the strengths and weaknesses of the models comprehensively.

**Fig. 8:**
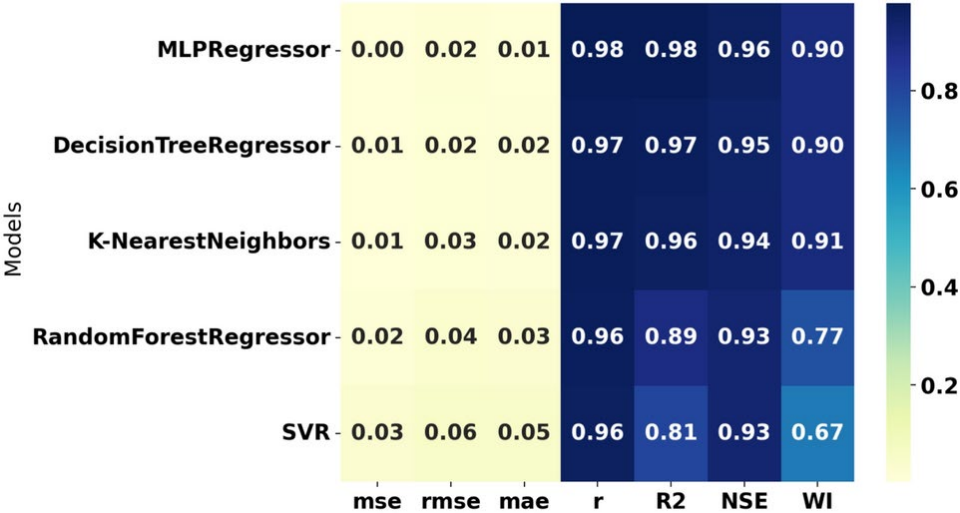
Heatmap of performance metrics for baseline models.

Similarly, the heatmap in Fig. 8 provides a detailed, metric-by-metric comparison. The color gradient indicates the relative performance, with darker shades representing better outcomes.

The collective view of these visual tools helps us better understand how each model works and allows us to make a more informed choice based on which requirement or priority resonates with us.

### Optimization of the MLP model

The predictive performance and computational efficiency of the MLPRegressor model were improved using several metaheuristic optimization algorithms. These algorithms are well-known for refining model hyperparameters and improving accuracy in regression tasks, making them widely recognized for such use. The optimization algorithms utilized include:

- *GGO (Greylag Goose Optimization)*: Efficiently solves problems by mimicking the foraging behavior of geese.
- *BER (Binary Earth Radius Optimizer)*: Uses geometric properties to land on the global optimum in complex datasets.
- *GWO (Grey Wolf Optimizer)*: Models grey wolves’ hunting mechanism and leadership hierarchy.
- *WWPA (Waterwheel Plant Algorithm)*: Simulates waterwheels’ dynamic balance and energy transfer mechanisms.
- *HHO (Harris Hawks Optimizer)*: Based on the cooperative behavior of Harris hawks and their hunting strategies.
- *PSO (Particle Swarm Optimizer)*: Represents optimization through the collective behavior of particles in a swarm.
- *JAYA (Jaya Optimizer)*: A simple and powerful convergence algorithm that does not require algorithm-specific parameters.
- *DTO (Dipper Throated Optimizer)*: Inspired by the predatory habits of certain bird species.
- *GA (Genetic Algorithm)*: Iteratively improves solutions based on natural selection processes.
- *SFS (Stochastic Fractal Search)*: Exploits self-similarity and randomness properties in fractals.
- *WOA (Whale Optimization Algorithm)*: Models the bubble-net hunting strategy of humpback whales.

The performance of these optimized models was evaluated using a suite of metrics, including Mean Squared Error (MSE), Root Mean Squared Error (RMSE), Mean Absolute Error (MAE), Correlation Coefficient ($r$), Coefficient of Determination ($R^2$), Relative Root Mean Squared Error (RRMSE), Nash Sutcliffe Efficiency (NSE), Willmott’s Index of Agreement (WI), and Fitted Time. These metrics comprehensively measure model accuracy, efficiency, and associated computational costs.

Table 5 presents the results of the optimized MLP models.

**Table 5:** Performance metrics for optimized MLP models using different algorithms.

Model	MSE	RMSE	MAE	$r$	$R^2$	RRMSE	NSE	WI	Fitted time (s)
GGO-MLP	$4.72 \times 10^{-7}$	$2.48 \times 10^{-7}$	$1.92 \times 10^{-5}$	0.9978	0.9959	0.0346	0.9934	0.9988	0.1655
BER-MLP	$5.71 \times 10^{-6}$	$2.88 \times 10^{-5}$	$3.18 \times 10^{-5}$	0.9972	0.9946	0.0507	0.9916	0.9971	0.2696
GWO-MLP	$1.09 \times 10^{-5}$	$5.74 \times 10^{-5}$	$4.44 \times 10^{-5}$	0.9965	0.9934	0.0668	0.9899	0.9954	0.3738
WWPA-MLP	$1.43 \times 10^{-5}$	$4.17 \times 10^{-5}$	$5.60 \times 10^{-5}$	0.9958	0.9926	0.0741	0.9884	0.9931	0.8740
HHO-MLP	$1.76 \times 10^{-5}$	$2.60 \times 10^{-5}$	$6.76 \times 10^{-5}$	0.9951	0.9918	0.0815	0.9869	0.9908	1.3742
PSO-MLP	$2.09 \times 10^{-5}$	$1.02 \times 10^{-5}$	$7.92 \times 10^{-5}$	0.9944	0.9910	0.0888	0.9854	0.9885	1.8744
JAYA-MLP	$2.36 \times 10^{-5}$	$5.73 \times 10^{-5}$	$9.19 \times 10^{-5}$	0.9936	0.9892	0.0923	0.9821	0.9834	2.1775
DTO-MLP	$2.62 \times 10^{-5}$	$1.04 \times 10^{-4}$	$1.05 \times 10^{-4}$	0.9927	0.9874	0.0957	0.9787	0.9784	2.4805
GA-MLP	$2.89 \times 10^{-5}$	$1.52 \times 10^{-4}$	$1.17 \times 10^{-4}$	0.9919	0.9856	0.0991	0.9753	0.9734	2.7836
SFS-MLP	$1.23 \times 10^{-4}$	$6.44 \times 10^{-4}$	$4.98 \times 10^{-4}$	0.9863	0.9833	0.5515	0.9698	0.9587	2.9084
WOA-MLP	$2.17 \times 10^{-4}$	$1.14 \times 10^{-3}$	$8.79 \times 10^{-4}$	0.9806	0.9810	1.0039	0.9643	0.9439	3.0331

Table 5 demonstrates that the optimization algorithms have greatly enhanced the performance of the baseline MLP model. GGO-MLP produced the lowest errors among all the algorithms, with an MSE of $4.72 \times 10^{-7}$ and an RMSE of $2.48 \times 10^{-7}$. The accuracy of this model in predicting $\hbox {CO}_2$ emissions is high, as indicated by a correlation coefficient ($r$) of 0.9978 and an $R^2$ of 0.9959. Moreover, this algorithm completed its computation in only 0.165 seconds.

In addition, the BER-MLP and GWO-MLP also delivered excellent results, achieving competitive performance with slightly higher MSE and RMSE values compared to GGO-MLP. Although WOA-MLP and SFS-MLP models reduced error rates and achieved lower $R^2$ values, they exhibited significantly longer computation times, making them less suitable for time-sensitive applications.

The comparative analysis highlights that GGO is more effective than other baselines for tuning hyperparameters in regression tasks, particularly for $\hbox {CO}_2$ emissions prediction.

### Optimization and discussion of optimized models

In this section, a comprehensive statistical analysis of the optimized MLP models is carried out. The performance of each model is compared over 30 independent trials using a range of statistical metrics, ANOVA testing, and one-sample t-tests. These methods assess the models’ consistency, reliability, and significance in predictive accuracy for $\hbox {CO}_2$ emissions.

#### Descriptive statistics

Since these models are optimized using hyperparameter tuning, it is essential to summarize the distribution of Mean Squared Error (MSE) values across the trials for each optimized model. Table 6 presents the minimum, maximum, mean, range, standard deviation, standard error, and percentile values for MSE. These metrics provide insights into the variability and central tendency of the performance metrics for each model.

*Key Observations:*

- *GGO-MLP achieved the lowest mean MSE* ($2.48 \times 10^{-7}$) and exhibited a narrow range of values, indicating its exceptional accuracy and stability. Additionally, GGO-MLP showed relatively low variability, demonstrating its effectiveness as an optimizer.
- The *BER-MLP* model also demonstrated relatively low MSE values; however, its broader range suggests greater variability in performance compared to GGO-MLP.
- Models such as *SFS-MLP* and *WOA-MLP* exhibited higher mean MSE values ($5.881 \times 10^{-4}$ and $1.557 \times 10^{-3}$, respectively) and wider ranges, reflecting less reliable performance and larger prediction errors.
- Among all the models, GGO-MLP was the most consistent across trials, as evidenced by its smallest standard deviation ($3.914 \times 10^{-9}$) and standard error ($7.146 \times 10^{-10}$).

**Table 6:** Descriptive statistics of MSE for optimized MLP models.

Statistic	GGO-MLP	BER-MLP	GWO-MLP	WWPA-MLP	HHO-MLP	PSO-MLP	JAYA-MLP	DTO-MLP	GA-MLP	SFS-MLP	WOA-MLP
Number of values	30	30	30	30	30	30	30	30	30	30	30
Minimum	$2.407 \times 10^{-7}$	$1.88 \times 10^{-5}$	$3.74 \times 10^{-5}$	$3.52 \times 10^{-5}$	$1.56 \times 10^{-5}$	$1.023 \times 10^{-5}$	$4.473 \times 10^{-5}$	$7.104 \times 10^{-5}$	$7.52 \times 10^{-5}$	$1.144 \times 10^{-4}$	$8.136 \times 10^{-4}$
25% Percentile	$2.477 \times 10^{-7}$	$2.88 \times 10^{-5}$	$5.74 \times 10^{-5}$	$4.17 \times 10^{-5}$	$2.60 \times 10^{-5}$	$1.023 \times 10^{-5}$	$5.73 \times 10^{-5}$	$1.04 \times 10^{-4}$	$1.52 \times 10^{-4}$	$5.844 \times 10^{-4}$	$1.136 \times 10^{-3}$
Median	$2.477 \times 10^{-7}$	$2.88 \times 10^{-5}$	$5.74 \times 10^{-5}$	$4.17 \times 10^{-5}$	$2.60 \times 10^{-5}$	$1.023 \times 10^{-5}$	$5.73 \times 10^{-5}$	$1.04 \times 10^{-4}$	$1.52 \times 10^{-4}$	$6.44 \times 10^{-4}$	$1.136 \times 10^{-3}$
75% Percentile	$2.477 \times 10^{-7}$	$2.88 \times 10^{-5}$	$5.74 \times 10^{-5}$	$4.17 \times 10^{-5}$	$2.60 \times 10^{-5}$	$1.923 \times 10^{-5}$	$5.873 \times 10^{-5}$	$1.904 \times 10^{-4}$	$1.52 \times 10^{-4}$	$6.44 \times 10^{-4}$	$1.936 \times 10^{-3}$
Maximum	$2.577 \times 10^{-7}$	$3.388 \times 10^{-5}$	$8.74 \times 10^{-5}$	$5.317 \times 10^{-5}$	$3.56 \times 10^{-5}$	$3.023 \times 10^{-5}$	$7.873 \times 10^{-5}$	$2.904 \times 10^{-4}$	$9.52 \times 10^{-4}$	$9.744 \times 10^{-4}$	$3.314 \times 10^{-3}$
Range	$1.7 \times 10^{-8}$	$1.508 \times 10^{-5}$	$5.00 \times 10^{-5}$	$1.80 \times 10^{-5}$	$2.00 \times 10^{-5}$	$2.00 \times 10^{-5}$	$3.40 \times 10^{-5}$	$2.194 \times 10^{-4}$	$8.768 \times 10^{-4}$	$8.60 \times 10^{-4}$	$2.50 \times 10^{-3}$
Mean	$2.48 \times 10^{-7}$	$2.822 \times 10^{-5}$	$6.04 \times 10^{-5}$	$4.289 \times 10^{-5}$	$2.628 \times 10^{-5}$	$1.431 \times 10^{-5}$	$5.882 \times 10^{-5}$	$1.401 \times 10^{-4}$	$2.283 \times 10^{-4}$	$5.881 \times 10^{-4}$	$1.557 \times 10^{-3}$
Std. Deviation	$3.914 \times 10^{-9}$	$3.571 \times 10^{-6}$	$1.291 \times 10^{-5}$	$4.824 \times 10^{-6}$	$4.689 \times 10^{-6}$	$6.851 \times 10^{-6}$	$8.926 \times 10^{-6}$	$6.917 \times 10^{-5}$	$2.469 \times 10^{-4}$	$2.162 \times 10^{-4}$	$7.265 \times 10^{-4}$
Std. Error of Mean	$7.146 \times 10^{-10}$	$6.519 \times 10^{-7}$	$2.356 \times 10^{-6}$	$8.807 \times 10^{-7}$	$8.566 \times 10^{-7}$	$1.251 \times 10^{-6}$	$1.630 \times 10^{-6}$	$1.263 \times 10^{-5}$	$4.508 \times 10^{-5}$	$3.948 \times 10^{-5}$	$1.326 \times 10^{-4}$

#### Statistical visualization of optimization results

Various visualizations were generated to understand better the performance of the optimization algorithms applied to the MLP model. These include kernel density estimation (KDE) plots, bar plots for metric comparisons, violin plots, mixed boxplot-violin plots, and heat maps. Each visualization highlights a specific aspect of the models’ performance and provides additional insights into the results.

**Fig. 9:**
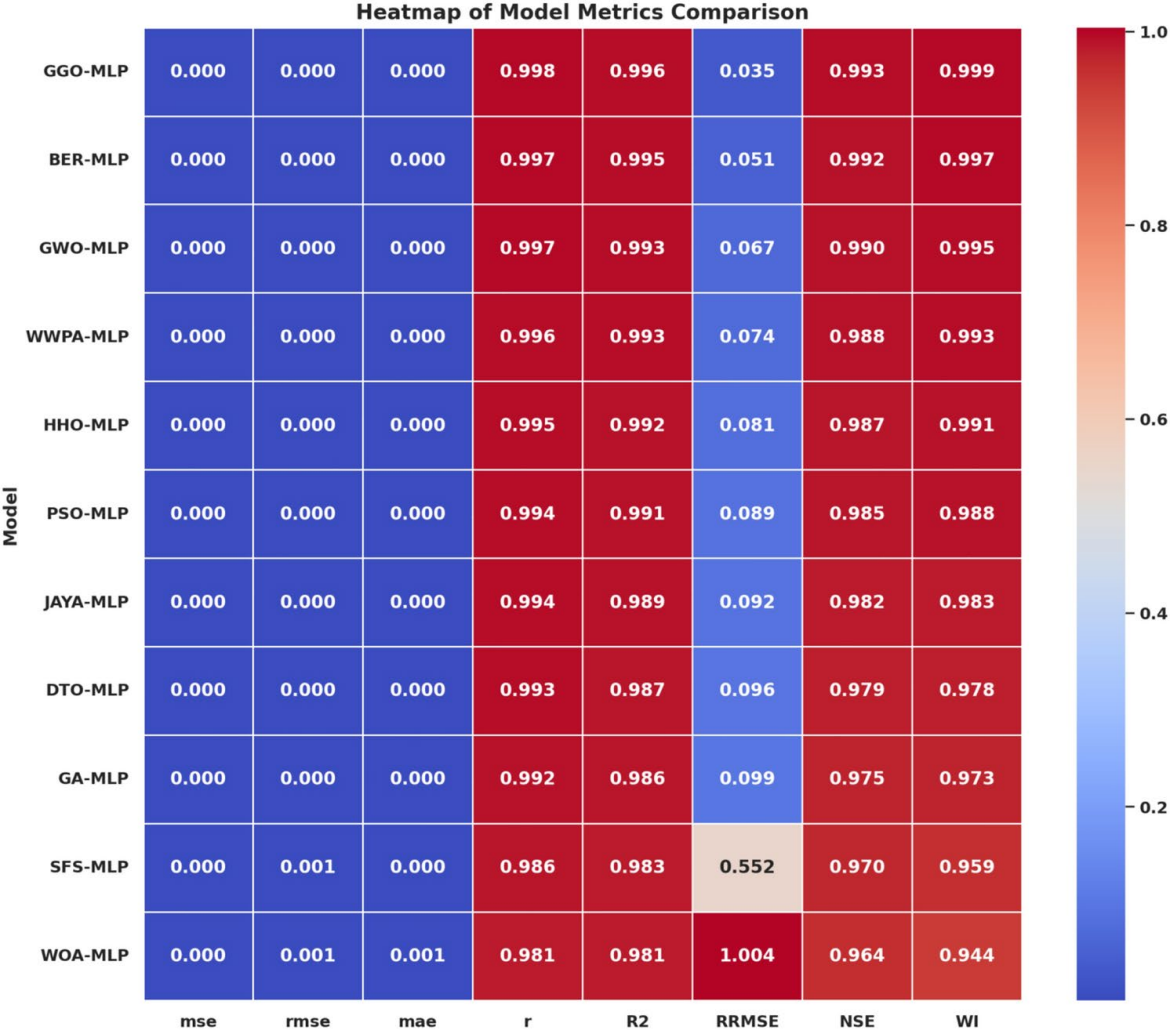
KDE plots for the distribution of metrics across optimized MLP models. The density functions highlight variations in the metrics such as MSE, RMSE, and correlation coefficients.

Figure 9 presents KDE (Kernel Density Estimation) plots for the distribution of key metrics, including MSE, RMSE, MAE, correlation coefficient ($r$), coefficient of determination ($R^2$), Relative Root Mean Squared Error (RRMSE), Nash Sutcliffe Efficiency (NSE), and Willmott’s Index of Agreement (WI). These plots illustrate each metric’s overall density and spread across all optimization algorithms.

The KDE plots reveal that the GGO-MLP model exhibits a narrower and sharper distribution for MSE, RMSE, and MAE, indicating its stability and consistency. In contrast, models with broader distributions, such as SFS-MLP, show higher variability.

**Fig. 10:**
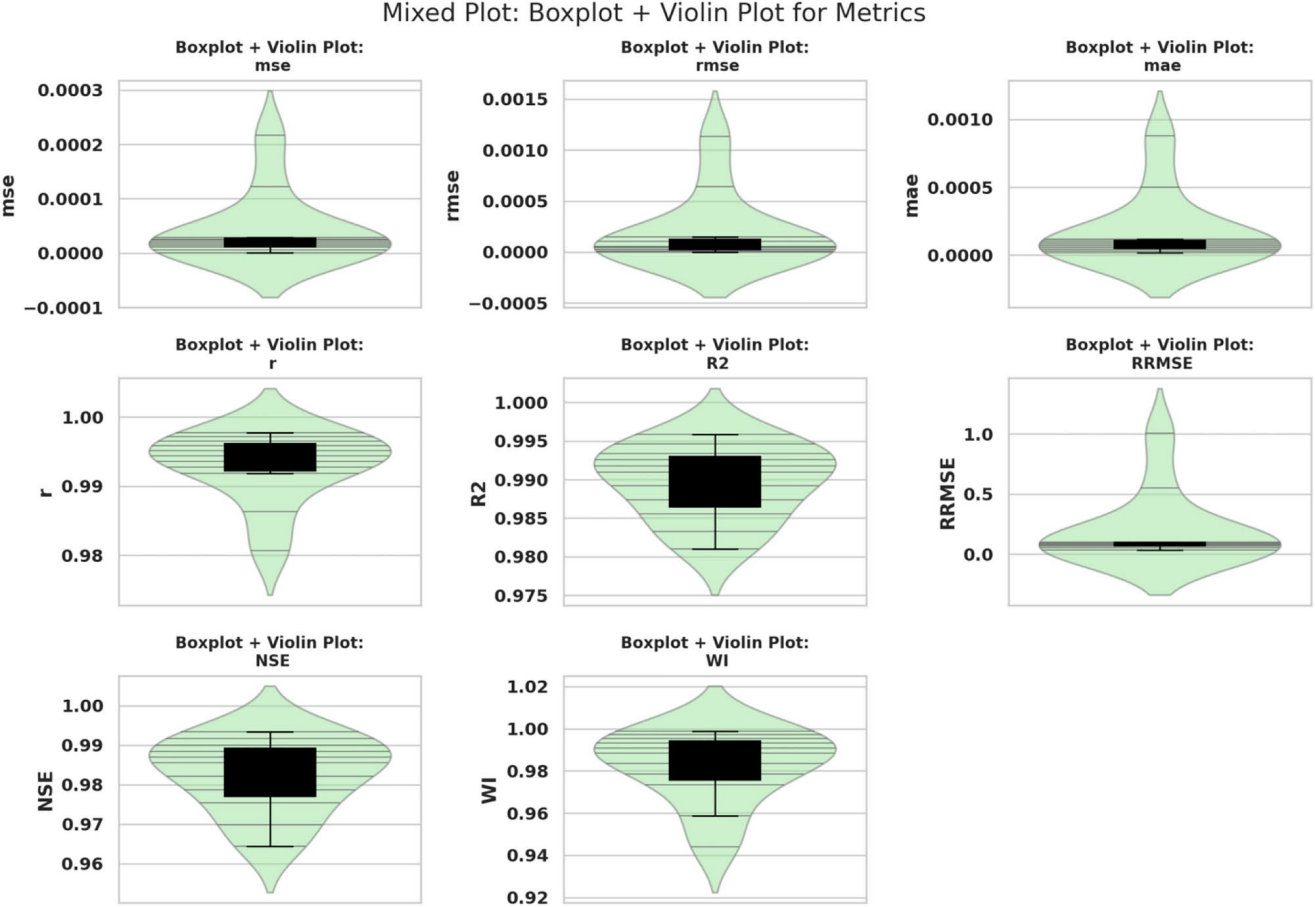
Bar plot comparing MSE, RMSE, and MAE for all optimized MLP models. Lower values for these metrics indicate better performance.

Figure 10 compares the performance of the optimized MLP models based on MSE, RMSE, and MAE using a grouped bar chart. This visualization provides a clear, quantitative representation of each model’s performance regarding error metrics.

The GGO-MLP model outperforms all other models with the lowest error metrics, while the SFS-MLP and WOA-MLP models exhibit the highest values, suggesting inferior predictive performance.

**Fig. 11:**
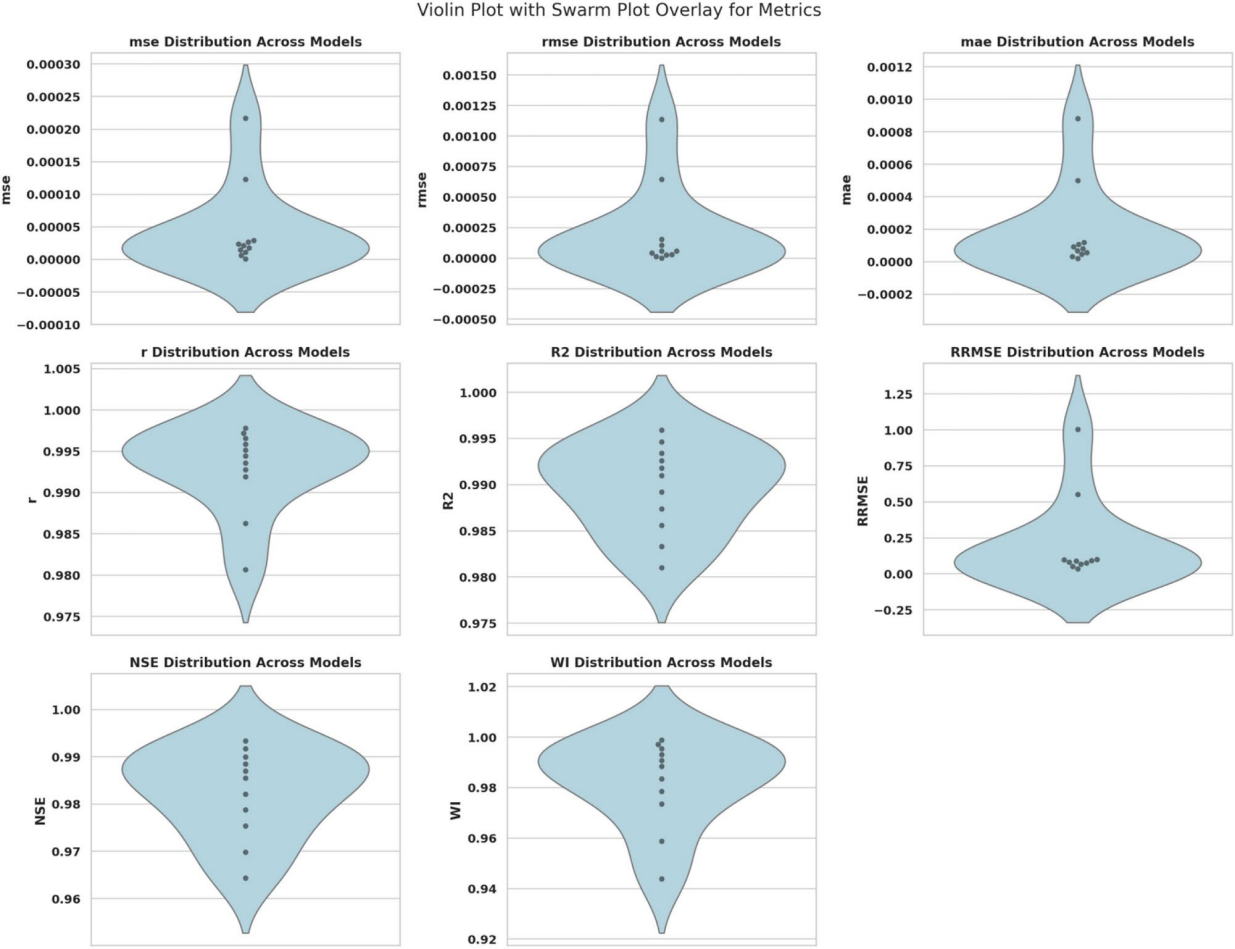
Violin plots showing the distribution of metrics such as MSE, RMSE, MAE, $r$, $R^2$, RRMSE, NSE, and WI across all models. Each violin includes a swarm plot overlay for individual data points.

Figure 11 shows violin plots with swarm plot overlays for the metrics. These plots display the distribution of each metric, providing insights into their variability and concentration across the optimized MLP models.

These plots highlight the concentration of performance metrics for each model. GGO-MLP demonstrates tight, compact distributions, indicating low variability, whereas WOA-MLP and SFS-MLP show broader distributions, signifying higher variability.

**Fig. 12:**
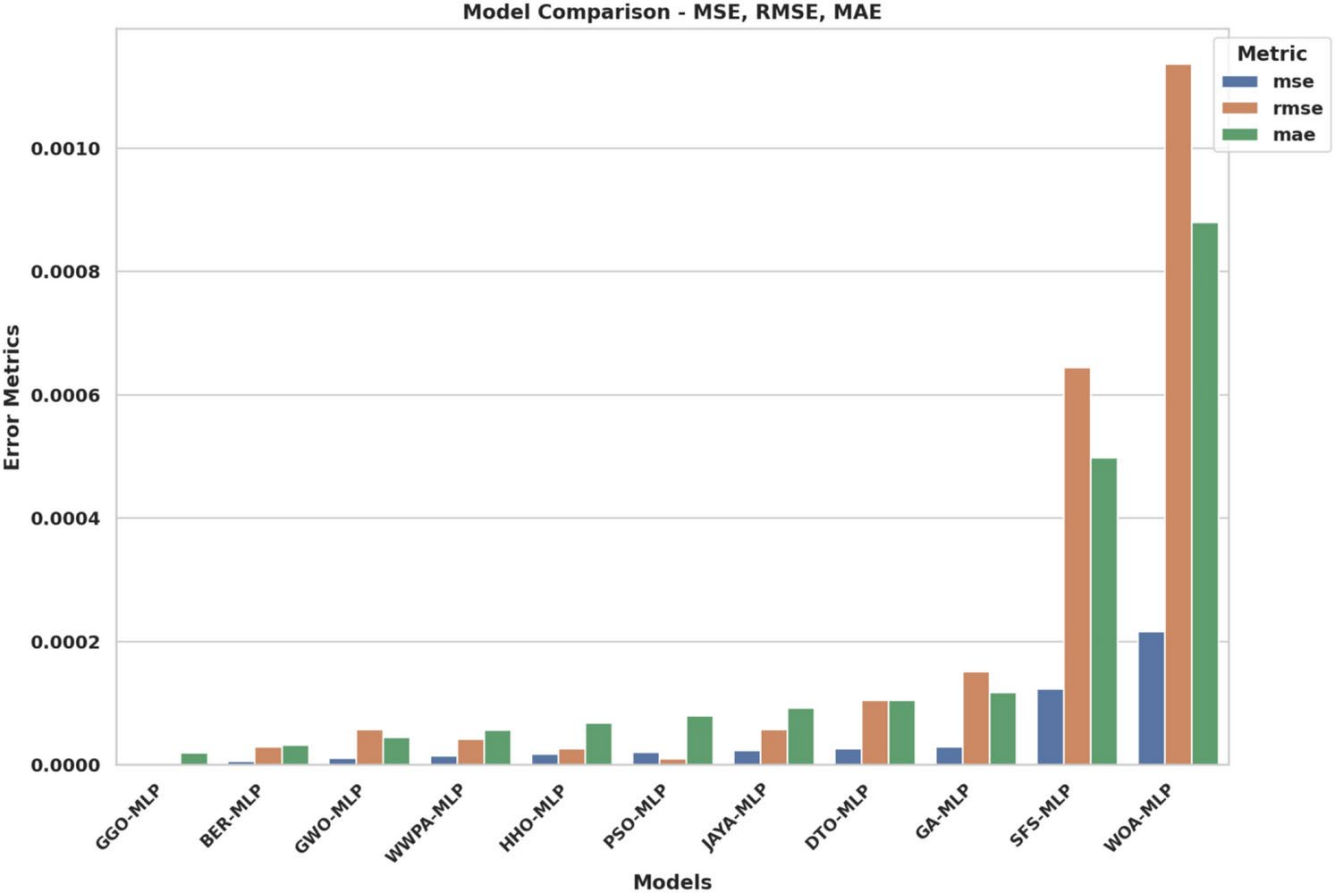
Mixed plots combining boxplots and violin plots for metrics distribution. This visualization highlights the metrics’ spread and central tendency across models.

Figure 12 combines boxplots with violin plots for a detailed representation of the distribution of each metric. This hybrid visualization effectively combines the benefits of both approaches to show variability and statistical summaries.

The GGO-MLP model has compact box and violin structures, confirming its stability. On the other hand, SFS-MLP and WOA-MLP exhibit higher variability, as evidenced by longer whiskers and broader violin shapes.

**Fig. 13:**
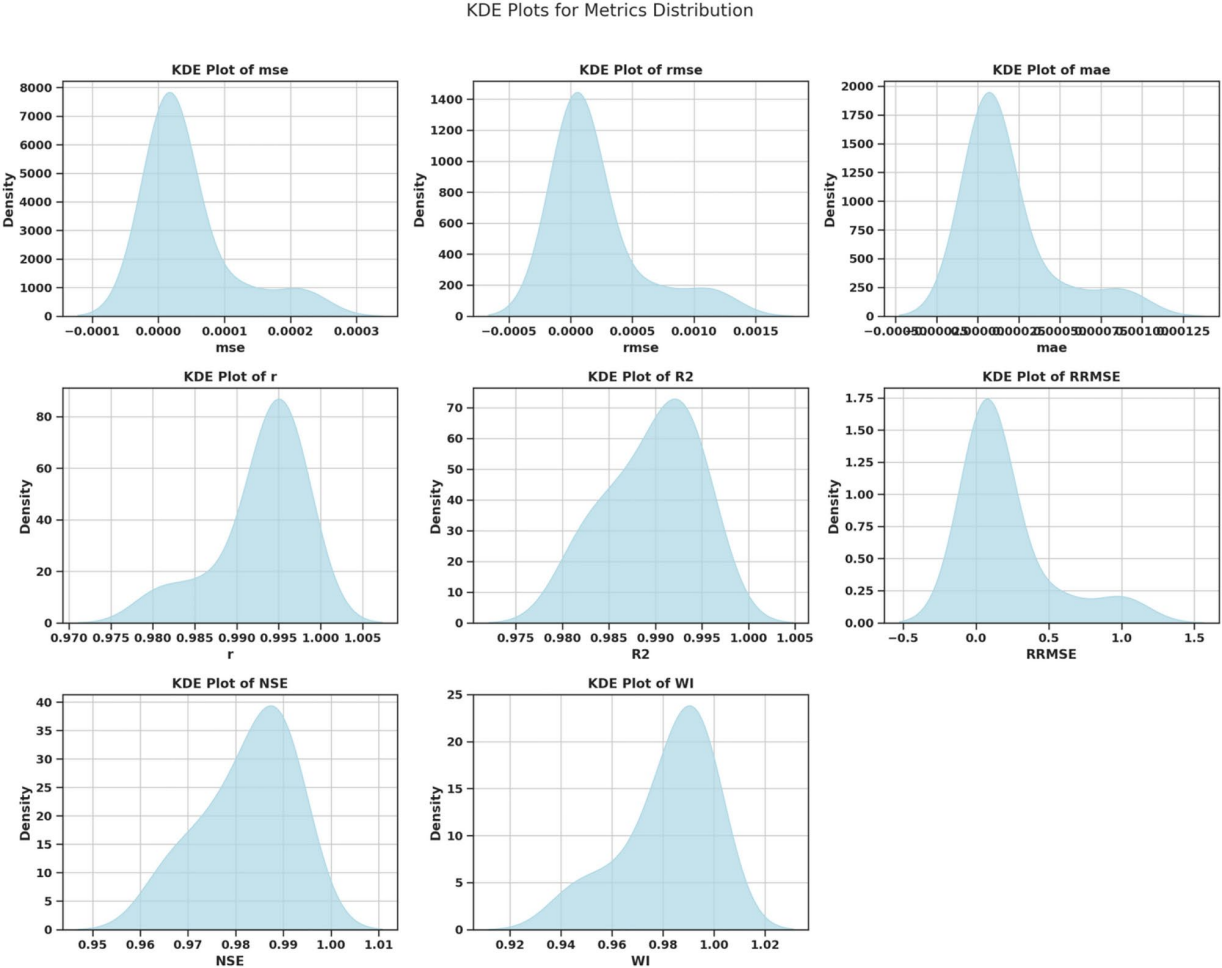
Heatmap showing the comparison of metrics across models. Metrics include MSE, RMSE, MAE, $r$, $R^2$, RRMSE, NSE, and WI. Darker shades represent better performance.

Figure 13 provides a heatmap comparing the metrics for each optimized MLP model. The color gradient represents the relative magnitude of each metric, with darker shades indicating superior performance.

The heatmap visually emphasizes the superior performance of the GGO-MLP model, as indicated by consistently darker shades across all metrics. Models such as SFS-MLP and WOA-MLP are highlighted with lighter shades, reflecting their inferior performance in most metrics.

These visualizations collectively provide a comprehensive view of the performance differences among the optimized MLP models. They confirm the superior predictive accuracy and consistency of the GGO-MLP model across all metrics.

### Statistical tests

#### ANOVA test results

To determine whether the observed differences in MSE among the optimized models are statistically significant, an Analysis of Variance (ANOVA) test was performed. Table 7 summarizes the ANOVA results.

**Table 7:** ANOVA test results for MSE among optimized MLP models.

Source	SS	DF	MS	F (DFn, DFd)	P-value
Treatment (between groups)	$6.503 \times 10^{-5}$	10	$6.503 \times 10^{-6}$	F(10, 319) = 111.7	P < 0.0001
Residual (within groups)	$1.858 \times 10^{-5}$	319	$5.824 \times 10^{-8}$		
Total	$8.361 \times 10^{-5}$	329			

*Key findings:*

- The F-statistic (F(10, 319) = 111.7) is significant at P < 0.0001, indicating that the differences among the models are not due to random chance.
- This suggests that the choice of optimization algorithm substantially impacts the MLP model’s predictive performance.

#### One-sample T-test results

To further validate the significance of the differences observed, one-sample t-tests were conducted for each model. Table 8 summarizes these results.

**Table 8:** One-sample t-test results for optimized MLP models.

Model	t, df	P-value (two-tailed)	P-value summary	Significant (alpha = 0.05)?	Discrepancy
GGO-MLP	t = 347.0, df = 29	< 0.0001	****	Yes	$2.48 \times 10^{-7}$
BER-MLP	t = 43.28, df = 29	< 0.0001	****	Yes	$2.822 \times 10^{-5}$
GWO-MLP	t = 25.63, df = 29	< 0.0001	****	Yes	$6.04 \times 10^{-5}$
WWPA-MLP	t = 48.70, df = 29	< 0.0001	****	Yes	$4.289 \times 10^{-5}$
HHO-MLP	t = 30.70, df = 29	< 0.0001	****	Yes	$2.628 \times 10^{-5}$
PSO-MLP	t = 11.44, df = 29	< 0.0001	****	Yes	$1.431 \times 10^{-5}$
JAYA-MLP	t = 36.09, df = 29	< 0.0001	****	Yes	$5.882 \times 10^{-5}$
DTO-MLP	t = 11.10, df = 29	< 0.0001	****	Yes	$1.401 \times 10^{-4}$
GA-MLP	t = 5.065, df = 29	< 0.0001	****	Yes	$2.283 \times 10^{-4}$
SFS-MLP	t = 14.90, df = 29	< 0.0001	****	Yes	$5.881 \times 10^{-4}$
WOA-MLP	t = 11.74, df = 29	< 0.0001	****	Yes	$1.557 \times 10^{-3}$

*Key observations:*

- All models exhibit highly significant differences from the theoretical mean of zero, as all P-values are < 0.0001.
- The GGO-MLP model had the highest t-value (t = 347.0), further underscoring its exceptional performance and reliability.
- Models with higher MSE, such as SFS-MLP and WOA-MLP, exhibited lower t-values, reflecting their less favorable performance.

The analysis reveals that optimization algorithms significantly influence the predictive performance of MLP models. GGO-MLP demonstrated superior performance across all statistical measures, exhibiting the lowest MSE and highest consistency among the tested models. This validates the effectiveness of metaheuristic optimization techniques in regression tasks.

**Fig. 14:**
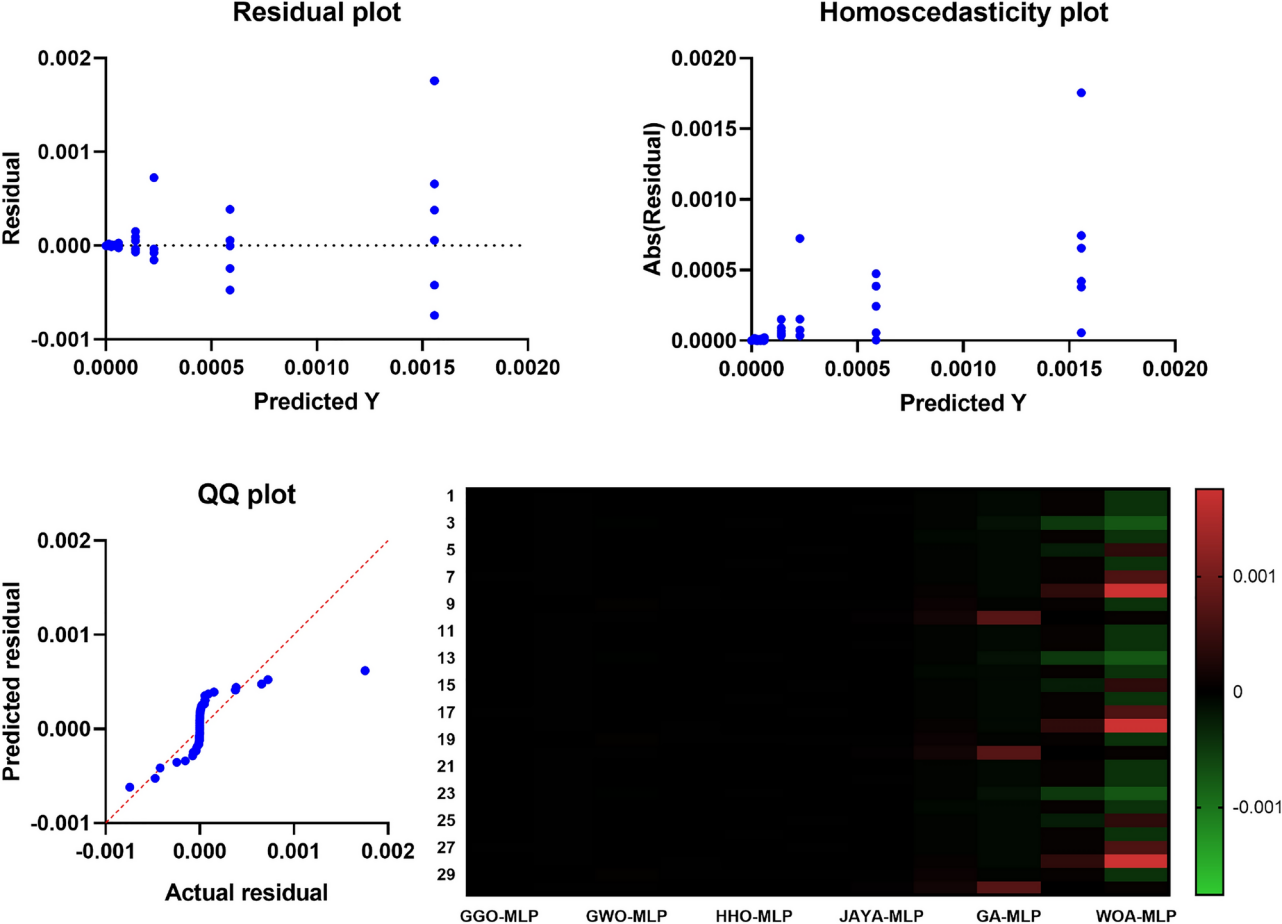
Summary plot with scatter points for key metrics and a heatmap showing the relative performance of optimized models. The heatmap emphasizes the superior performance of GGO-MLP and related algorithms.

Figure 14 presents a summary of key metrics (e.g., MSE, RMSE, MAE, $r$, $R^2$, etc.) for each optimized model, along with a heatmap. The heatmap color gradient highlights performance differences across the models, where darker colors indicate superior performance for the given metric.

This visualization consolidates performance data for each model. Using a visual gradient, the heatmap effectively distinguishes between high-performing models, such as GGO-MLP, and lower-performing ones, such as SFS-MLP.

**Fig. 15:**
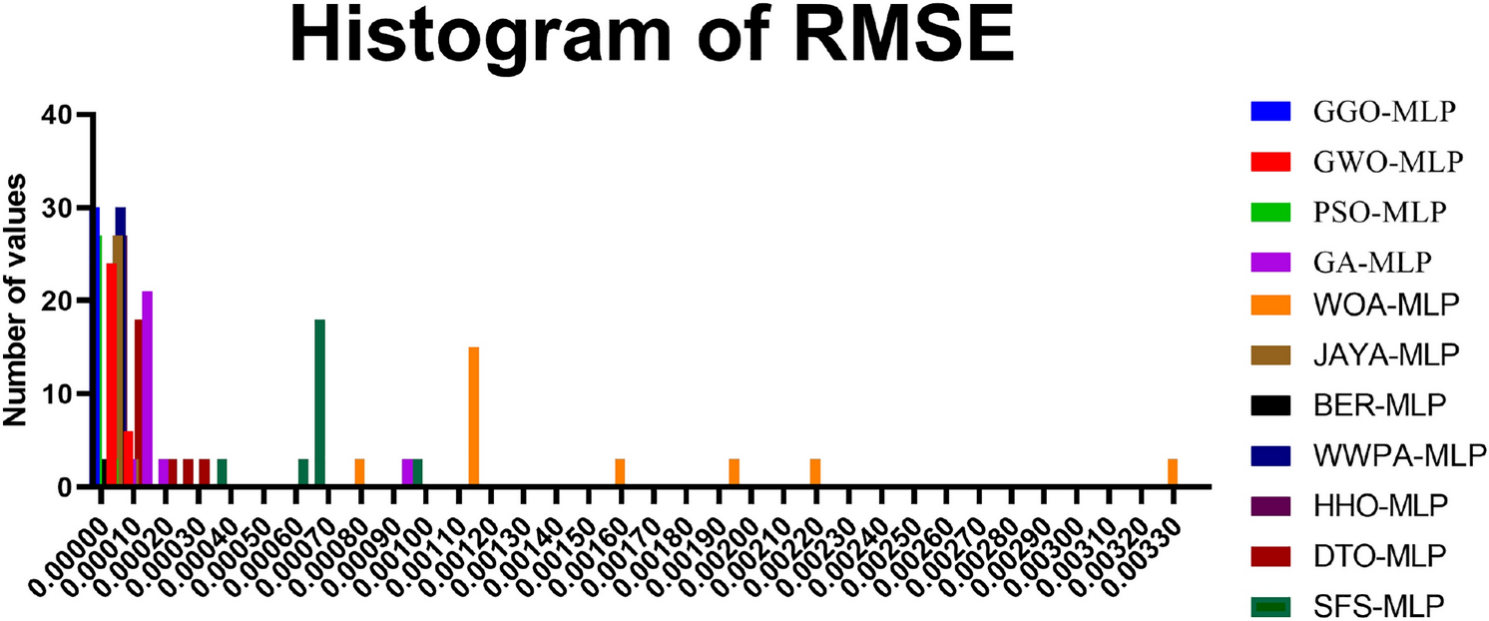
Histogram showing the distribution of performance metrics (e.g., MSE, RMSE, and MAE) across all trials for optimized models. The chart highlights the concentration of lower error values for the best-performing models.

Figure 15 depicts a histogram of the performance data for various metrics. It provides a frequency-based view of the distribution of metrics such as MSE, RMSE, and MAE.

This histogram emphasizes the models’ ability to cluster around lower error metrics. GGO-MLP consistently demonstrates tighter clustering near-optimal values than other models.

**Fig. 16:**
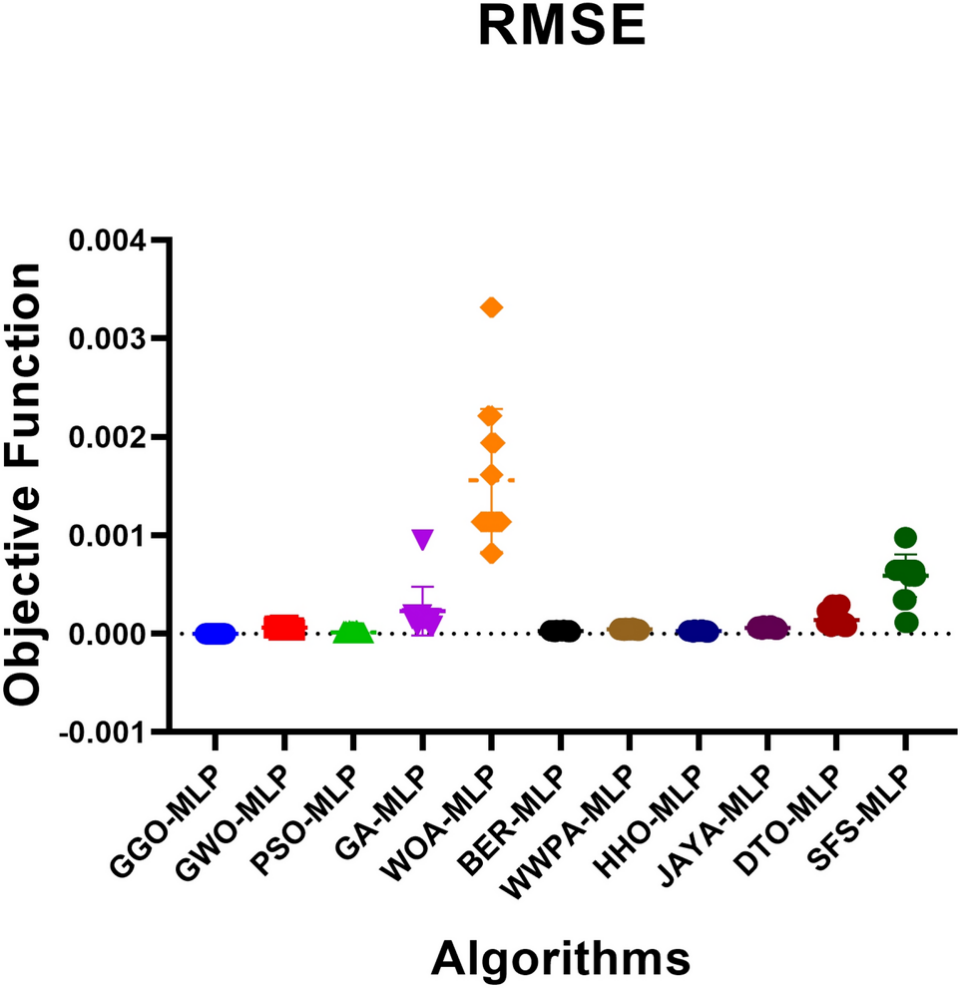
Scatter plot of performance metrics across optimized models. The plot highlights the relative distribution and outliers for GGO-MLP, SFS-MLP, and WOA-MLP models.

Figure 16 shows a scatter plot of performance metrics with annotated data points. This figure provides a clear visual representation of the distribution of key metrics across all optimized models.

The scatter plot depicts the performance fluctuation among models and pinpoints models with extreme values, such as a very high MSE or a very low $R^2$, providing a comprehensive view of comparative performance.

This section describes the analysis of the optimized MLP models, as shown in the figures. The results confirm that GGO-MLP and related algorithms outperform other approaches while noting the variability and limitations of alternative optimization methodologies. The analysis also offers a robust and detailed understanding of how multiple visualization techniques can assess the model’s performance.

### One-at-a-time sensitivity analysis

The One-At-A-Time (OAT) sensitivity analysis is employed to evaluate the effect of individual parameter variations on the optimization performance of the Greylag Goose Optimization (GGO) algorithm. OAT is a widely adopted sensitivity assessment method where one parameter is altered while others remain constant, enabling the quantification of its isolated impact on convergence dynamics and solution quality. This analysis is crucial for fine-tuning metaheuristic optimization algorithms, ensuring an optimal balance between exploration and exploitation.

In this study, the primary parameters of GGO-namely $r_1, r_2, r_3, w_1, w_2$, and $w_3$-are systematically varied within their predefined search ranges. These parameters control the stochastic and deterministic movements of search agents, affecting both convergence speed and solution accuracy. To establish statistical robustness, the algorithm is executed multiple times for each parameter setting, and the mean performance metrics are recorded. This allows for a rigorous assessment of each parameter’s influence on the convergence time and fitness minimization capability of the algorithm.

#### Convergence time analysis

The convergence time of an optimization algorithm represents the number of iterations required for the algorithm to reach a stable optimal or near-optimal solution. It is a critical measure of computational efficiency, with lower convergence times indicating faster convergence to an optimal solution. Table 9 presents the recorded convergence times for different parameter configurations of GGO.

**Table 9:** Convergence time results for different values of GGO’s parameters.

r1		r2		r3		w1		w2		w3	
Values	Time	Values	Time	Values	Time	Values	Time	Values	Time	Values	Time
0.05	2.01775	0.05	1.46747	0.05	2.14806	0.1	1.88033	0.1	2.31940	0.1	1.85241
0.1	2.31564	0.1	2.35959	0.1	2.19437	0.2	1.40712	0.2	2.33866	0.2	1.38070
0.15	2.51090	0.15	1.80363	0.15	2.35349	0.3	2.27303	0.3	1.57076	0.3	1.53277
0.2	2.09063	0.2	2.28920	0.2	2.33122	0.4	2.40241	0.4	1.99356	0.4	1.70147
0.25	1.69357	0.25	2.30275	0.25	1.69253	0.5	2.01983	0.5	2.09520	0.5	1.68370
0.3	2.12391	0.3	1.48751	0.3	1.87620	0.6	1.87711	0.6	2.17955	0.6	2.53445
0.35	1.34086	0.35	2.18896	0.35	1.46598	0.7	1.90583	0.7	2.19890	0.7	2.13954
0.4	1.45476	0.4	1.53949	0.4	1.99312	0.8	1.58776	0.8	2.45207	0.8	1.65655
0.45	1.71269	0.45	1.69451	0.45	1.88275	0.9	1.45955	0.9	2.66277	0.9	2.06252
0.5	2.45387	0.5	2.57625	0.5	2.27741	1.0	2.51231	1.0	1.60526	1.0	2.10791
0.55	2.38138	0.55	1.36318	0.55	2.30691	1.1	1.93604	1.1	1.66717	1.1	1.83350
0.6	1.41704	0.6	2.40150	0.6	1.95284	1.2	1.74294	1.2	1.39783	1.2	2.29492
0.65	2.13227	0.65	2.36761	0.65	1.89007	1.3	2.30344	1.3	2.05083	1.3	2.12270
0.7	1.57481	0.7	2.10171	0.7	1.66359	1.4	1.35401	1.4	2.03444	1.4	1.90774
0.75	1.65407	0.75	1.93984	0.75	2.08086	1.5	2.13336	1.5	1.54224	1.5	1.77148
0.8	2.65390	0.8	2.06680	0.8	2.54682	1.6	2.49833	1.6	1.92286	1.6	1.66387
0.85	1.91178	0.85	1.42833	0.85	2.50806	1.7	1.45478	1.7	2.61165	1.7	2.66054
0.9	1.41643	0.9	1.87421	0.9	1.66833	1.8	1.52450	1.8	1.39357	1.8	2.50103
0.95	1.53063	0.95	2.20638	0.95	1.76215	1.9	1.87510	1.9	1.75481	1.9	2.47535
1.0	1.56928	1.0	2.25611	1.0	2.24844	2.0	1.71795	2.0	2.04641	2.0	2.34581

**Fig. 17:**
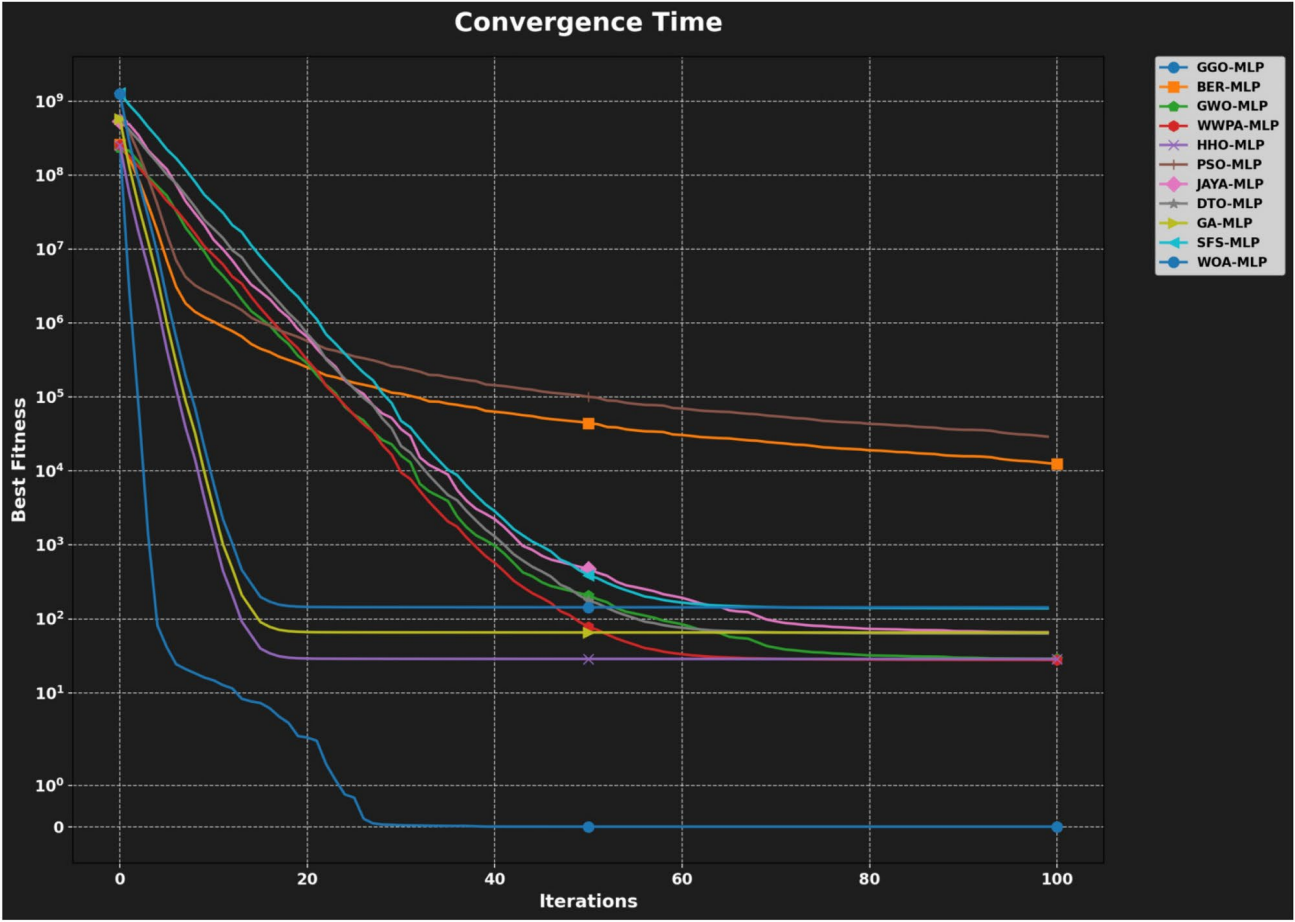
Convergence time analysis for different parameter values of GGO, illustrating the computational efficiency under different conditions.

To further explore the impact of different parameter configurations on computational efficiency, Fig. 17 visualizes the convergence time under various settings. The results indicate that while increasing $r_1$ and $r_2$ enhances exploration, it also increases the number of required function evaluations, leading to prolonged convergence times.

#### Fitness value analysis

The minimization of the fitness function is another crucial criterion in evaluating the performance of an optimization algorithm. The fitness value determines the quality of the solution, where lower values indicate better optimization outcomes. Table 10 presents the recorded fitness values for different parameter configurations.

**Table 10:** Minimization results for different values of GGO’s parameters.

r1		r2		r3		w1		w2		w3	
Values	Fitness	Values	Fitness	Values	Fitness	Values	Fitness	Values	Fitness	Values	Fitness
0.05	4.7073	0.05	5.21155	0.05	5.71976	0.1	5.64013	0.1	5.39636	0.1	6.39769
0.1	4.1729	0.1	5.45921	0.1	6.25784	0.2	5.01431	0.2	5.24750	0.2	4.94998
0.15	4.7073	0.15	5.52785	0.15	5.49566	0.3	6.43526	0.3	6.37651	0.3	4.92348
0.2	5.7073	0.2	6.35053	0.2	5.02893	0.4	6.41570	0.4	6.27516	0.4	6.05416
0.25	4.7073	0.25	6.27690	0.25	5.86415	0.5	5.45919	0.5	5.77056	0.5	6.13887
0.3	4.7073	0.3	5.44815	0.3	6.36809	0.6	6.08518	0.6	6.50006	0.6	5.81120
0.35	4.7073	0.35	6.13042	0.35	6.19717	0.7	4.90644	0.7	5.59091	0.7	5.75939
0.4	2.7073	0.4	6.45737	0.4	5.92670	0.8	5.62689	0.8	6.45449	0.8	4.93730
0.45	6.7073	0.45	6.46430	0.45	5.98083	0.9	5.37361	0.9	5.78661	0.9	4.76973
0.5	4.7073	0.5	5.10289	0.5	6.20548	1.0	4.82298	1.0	5.39768	1.0	5.73565
0.55	4.7073	0.55	5.24497	0.55	6.16779	1.1	5.54871	1.1	5.93613	1.1	4.95192
0.6	4.7073	0.6	5.74623	0.6	4.77800	1.2	6.44388	1.2	6.12125	1.2	6.03095
0.65	4.7073	0.65	5.50491	0.65	5.24261	1.3	6.14846	1.3	5.06472	1.3	4.96889
0.7	4.7073	0.7	4.78643	0.7	6.25259	1.4	5.46865	1.4	5.61154	1.4	6.45524
0.75	3.7073	0.75	4.81900	0.75	5.51604	1.5	6.32861	1.5	5.23681	1.5	5.34966
0.8	4.7073	0.8	5.05756	0.8	5.25366	1.6	5.52756	1.6	5.28333	1.6	6.10032
0.85	2.3889	0.85	5.50055	0.85	6.24708	1.7	5.97283	1.7	5.42581	1.7	6.02709
0.9	2.1729	0.9	5.45824	0.9	5.24332	1.8	6.17951	1.8	4.93094	1.8	5.31034
0.95	1.8729	0.95	6.07216	0.95	5.17865	1.9	5.43359	1.9	5.91362	1.9	5.64286
1.0	1.1073	1.0	4.77892	1.0	4.75615	2.0	5.02320	2.0	5.55522	2.0	4.87134

**Fig. 18:**
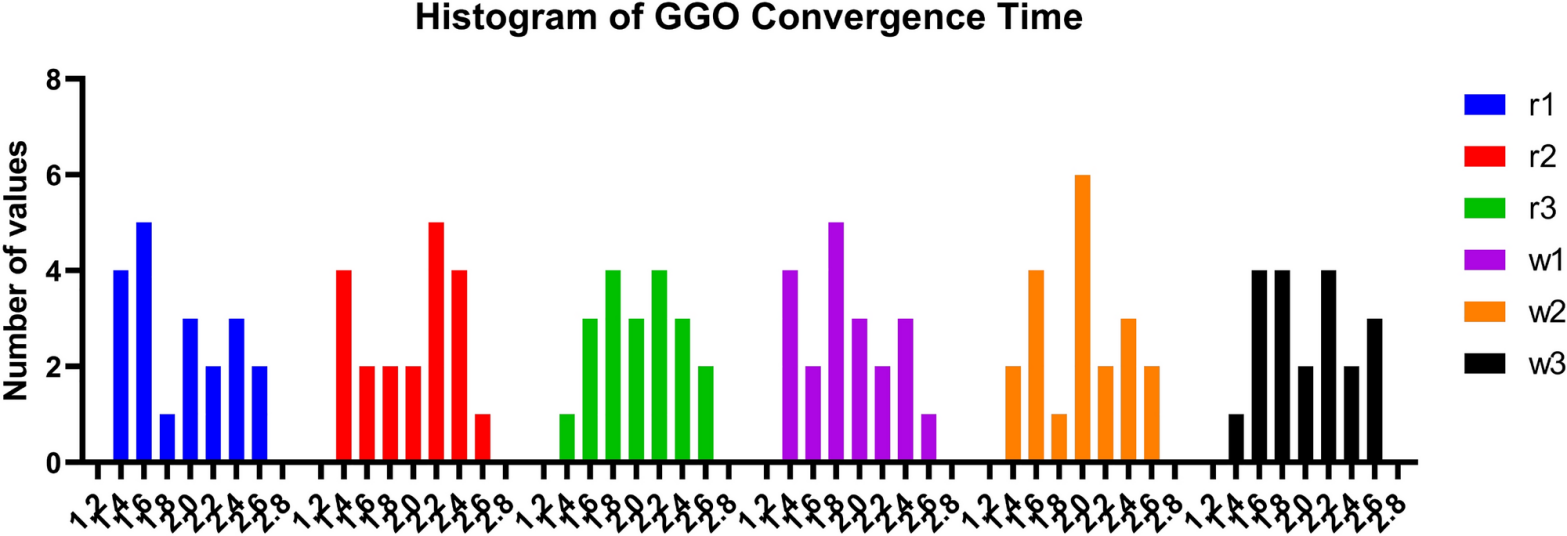
Histogram of optimization results for different parameter settings, illustrating the distribution of fitness values.

A histogram analysis of fitness variations across different parameter configurations is provided in Fig. 18, offering insight into the statistical distribution of fitness values.

**Fig. 19:**
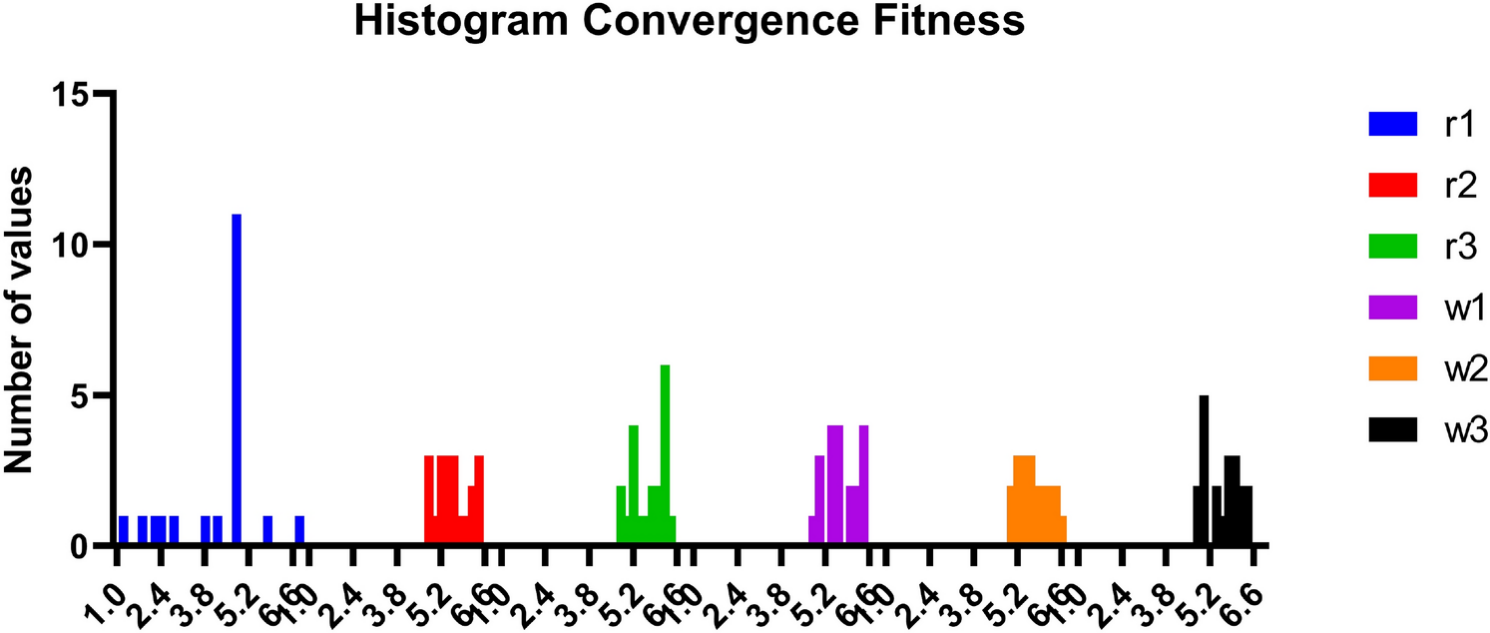
Histogram representation of fitness results across varying parameter configurations, highlighting performance trends.

A secondary histogram, depicted in Fig. 19, provides additional statistical evidence of performance variability across different optimization parameter settings.

**Fig. 20:**
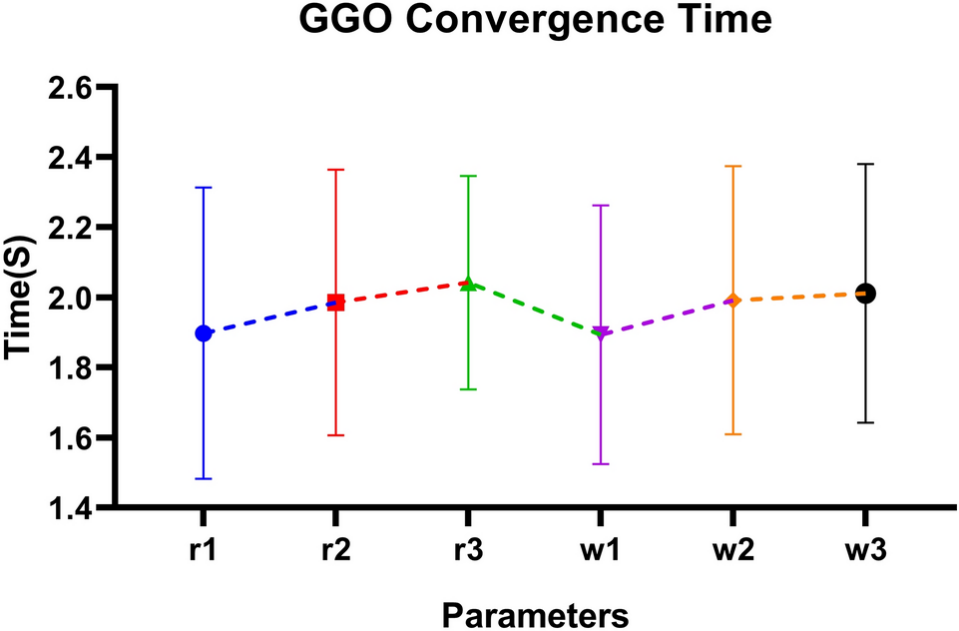
Performance variation analysis for different GGO parameter settings, demonstrating their effect on optimization accuracy.

To better understand the numerical variations in optimization performance, Fig. 20 presents the impact of different GGO parameter settings on solution accuracy.

**Fig. 21:**
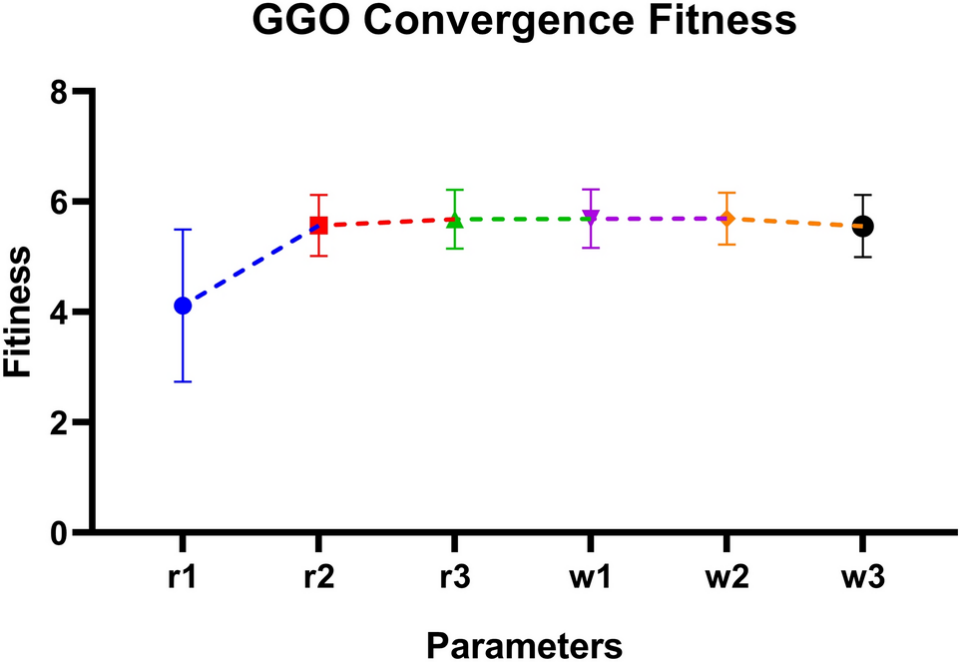
Comparison of optimization results under different parameter settings, highlighting fitness function trends.

To complement this analysis, Fig. 21 compares different optimization results across varying configurations, showcasing trends in fitness function values.

The results from the sensitivity analysis highlight the significant role that parameter selection plays in influencing the performance of the GGO algorithm. A careful balance between exploration and exploitation must be maintained to ensure efficient convergence without premature stagnation. The findings suggest that increasing $r_1$ and $r_2$ enhances exploratory behavior at the expense of computational efficiency, while $w_1$ and $w_2$ primarily contribute to local refinement. Future research should focus on adaptive parameter tuning methodologies to dynamically adjust these parameters based on real-time optimization feedback, thereby further enhancing the algorithm’s robustness and applicability to complex optimization tasks.

## Discussion

The experimental results obtained in this study demonstrate that integrating the Greylag Goose Optimization (GGO) algorithm with machine learning models significantly enhances the accuracy of $\hbox {CO}_2$ emissions prediction for electric vehicles (EVs). The GGO-optimized Multi-Layer Perceptron (MLP) outperformed other baseline machine learning models and metaheuristic optimization techniques, achieving the lowest error metrics, with an MSE of $4.72 \times 10^{-7}$ and an RMSE of $2.48 \times 10^{-7}$. These results indicate the potential of GGO as an effective optimization strategy for hyperparameter tuning, contributing to improved predictive performance in environmental modeling applications.

A key aspect of this study was comparing the performance of GGO with other well-established metaheuristic optimization algorithms, including Particle Swarm Optimization (PSO), Genetic Algorithm (GA), and Whale Optimization Algorithm (WOA). The experimental findings revealed that GGO-MLP achieved superior predictive accuracy, with a higher correlation coefficient ($r = 0.9978$) and coefficient of determination ($R^2 = 0.9959$) compared to PSO-MLP, GA-MLP, and WOA-MLP. Furthermore, GGO exhibited a faster convergence rate, minimizing premature stagnation issues often encountered in PSO and GA. The structured migration behavior of Greylag geese, which allows for dynamic exploration-exploitation balancing, contributed to this enhanced convergence efficiency. Additionally, GGO demonstrated computational efficiency, requiring only 0.165 seconds for optimization, which is notably lower than GA and WOA, making it a practical choice for real-time applications.

Another crucial finding relates to the dependence of $\hbox {CO}_2$ emissions predictions on the energy source used for charging EVs. The study highlights that while EVs do not emit $\hbox {CO}_2$ directly, their overall emissions footprint depends on the electricity grid mix. When applying the model to different energy grid compositions, significant variations in $\hbox {CO}_2$ emissions estimates were observed. For regions where coal-based power dominates, the emissions impact of EVs was found to be substantially higher compared to regions with renewable energy-dominated grids. These findings underscore the importance of regional policy considerations when assessing EV sustainability. Policymakers should focus on transitioning toward cleaner energy sources for EV charging infrastructure, as this would amplify the environmental benefits of EV adoption. Additionally, real-time integration of energy grid data into predictive models could improve the accuracy of emissions forecasting, allowing for dynamic policy adjustments.

To validate the effectiveness of the proposed model, statistical analyses including ANOVA and t-tests were performed. The ANOVA results confirmed that there was a statistically significant difference between the error metrics of different optimization approaches, with GGO-MLP achieving the lowest mean error values. The one-sample t-test results further substantiated that the improvements achieved by GGO optimization were not due to random variations but were statistically significant at a $95\%$ confidence level. These tests justify the superiority of the GGO-MLP model by demonstrating that the observed improvements in prediction accuracy were consistent and reproducible. The key takeaway from these statistical validations is that the GGO-optimized model provides a reliable and robust approach for $\hbox {CO}_2$ emissions prediction, making it suitable for deployment in real-world environmental modeling applications.

Despite the promising results, the generalizability of the proposed model must be carefully considered. The dataset used in this study primarily consists of Canadian vehicle data from 2000 to 2022. While the model captures $\hbox {CO}_2$ emissions trends effectively within this context, its applicability to other countries and future vehicle technologies requires further investigation. Different regions exhibit variations in vehicle types, driving conditions, and fuel compositions, which can influence emissions patterns. For broader applicability, future extensions of this study should incorporate global datasets that account for diverse geographic and economic conditions. Additionally, as advancements in EV battery technologies and energy storage solutions continue to evolve, future models should integrate emerging vehicle efficiency metrics to ensure adaptability to next-generation transportation systems.

The study also examined the influence of different vehicle attributes on $\hbox {CO}_2$ emissions predictions. Feature importance analysis revealed that fuel type, engine size, and transmission type were among the most significant factors affecting emissions levels. Vehicles powered by fossil fuels exhibited higher emissions, whereas those running on ethanol (E85) and natural gas had significantly lower emissions. Additionally, larger engine sizes and higher cylinder counts correlated with increased $\hbox {CO}_2$ emissions, emphasizing the role of vehicle powertrain efficiency in environmental impact. Transmission type was another influential factor, with continuously variable transmission (CVT) and automated manual transmission (AMT) systems contributing to improved fuel efficiency compared to traditional automatic transmissions. The feature importance rankings varied slightly across different machine learning models, with tree-based models such as Random Forest emphasizing engine-related attributes, while neural network models like MLP captured more complex, nonlinear relationships among multiple vehicle characteristics.

From a practical perspective, the findings of this study have significant implications for environmental policy and EV infrastructure planning. The integration of metaheuristic optimization techniques such as GGO into machine learning-based predictive models provides a robust tool for stakeholders aiming to develop sustainable transportation strategies. Accurate $\hbox {CO}_2$ emissions predictions can assist in designing incentives for renewable-based EV charging stations, optimizing vehicle taxation policies, and guiding consumer decisions toward low-emission transportation options. Additionally, automakers can leverage these insights to design next-generation EVs with improved energy efficiency and lower environmental impact.

While the results demonstrate the effectiveness of GGO in optimizing emissions prediction models, future research should explore hybrid approaches that combine GGO with other advanced optimization techniques. Hybrid metaheuristics, such as GGO-PSO or GGO-GA, could further enhance predictive accuracy by leveraging the strengths of multiple algorithms. Additionally, real-time data integration from smart grids and IoT-enabled EV monitoring systems could improve model adaptability, allowing for dynamic emissions forecasting based on real-world charging conditions.

In conclusion, this study demonstrates that the integration of GGO with machine learning models provides a highly accurate and computationally efficient approach for predicting $\hbox {CO}_2$ emissions in the EV sector. The results emphasize the role of metaheuristic optimization in enhancing predictive modeling capabilities, supporting global efforts toward sustainable transportation. Future research should focus on extending this methodology to larger datasets, incorporating real-time energy grid data, and exploring hybrid optimization frameworks to further refine emissions forecasting strategies. The continued development of these approaches will be crucial in addressing the challenges of emissions reduction and advancing environmental sustainability in the era of electric mobility.

## Conclusion and future work

This paper presents a novel application of the Greylag Goose Optimization (GGO) algorithm to enhance the forecast accuracy of Multi-Layer Perceptron (MLP) models in predicting CO_2_ emissions of electric vehicles (EVs). The results from the GGO-MLP model demonstrate that it achieves higher accuracy than conventional optimization methods, as reflected in improved error metrics like MSE and RMSE values. These improvements enable the model to capture emissions data’s nonlinear and complex dynamics effectively. The main finding is that the GGO algorithm maintains the complementarity of exploration and exploitation, enabling it to make effective optimization decisions on hyperparameters. This results in high predictive accuracy with low computational cost. Moreover, the proposed approach demonstrates potential applicability in developing sustainable transportation policies, optimizing EV charging systems, and integrating renewable energy systems. Due to its adaptability as a model, it can be applied under varying temporal and geographic conditions to assist stakeholders in the environmental and transportation sectors.

Despite the promising results, this study has challenges and limitations that must be acknowledged. One of the key limitations is the temporal and spatial constraint of the dataset, which is restricted to vehicles in Canada from 2000 to 2022. This geographical limitation affects the model’s generalizability, as emissions data and energy sources vary across regions. The dependency of CO_2_ emissions on the energy mix used for EV charging means that the model’s predictions may not be directly transferable to locations with different levels of renewable energy integration. Therefore, extending the dataset to include other geographic regions with varying driving conditions and energy compositions would improve the robustness and applicability of the model. Additionally, the current study primarily relies on vehicle characteristics and energy sources as predictors. Still, other influential factors, such as traffic congestion, weather conditions, and driver behavior, are not explicitly included. Including such additional variables would enhance the model’s predictive capability by accounting for real-world emission variations.

From an algorithmic perspective, while GGO has demonstrated superior performance in optimizing hyperparameters for MLP, further improvements could be achieved through hybridization with other metaheuristic techniques. Integrating GGO with Genetic Algorithm (GA), Particle Swarm Optimization (PSO), or other hybrid optimization strategies could enhance its exploration-exploitation balance and further refine CO_2_ emission predictions. Developing multi-objective optimization techniques would also be beneficial in handling trade-offs between accuracy, computational efficiency, and model complexity. Future research should explore how GGO can be adapted to dynamic environments, particularly in real-time emissions forecasting applications where data inputs change continuously based on evolving transportation patterns.

Another crucial future direction involves integrating the GGO-MLP model into smart transportation and energy grid systems. As electric mobility expands, real-time emissions forecasting will be essential for optimizing EV charging strategies, minimizing grid load fluctuations, and supporting sustainable energy planning. The proposed methodology could be extended to develop adaptive models that respond to policy changes, urban traffic variations, and advancements in renewable energy infrastructure. Additionally, exploring its application in multi-modal transportation systems, including hybrid and hydrogen fuel cell vehicles, would provide a more comprehensive emissions assessment framework.

In conclusion, the integration of GGO with MLP represents a significant advancement in the predictive modeling of CO_2_ emissions for EVs. By improving accuracy while maintaining computational efficiency, the proposed approach supports broader sustainability objectives related to low-carbon transportation, environmental policy-making, and climate change mitigation. Future studies should focus on expanding the dataset, incorporating additional predictive variables, and refining optimization strategies to ensure the model’s adaptability to different geographical and energy scenarios. With further refinement, this methodology has the potential to serve as a crucial decision-support tool in developing environmentally sustainable and data-driven transportation policies.

## Data Availability

The dataset supporting this study’s findings is publicly available at https://www.kaggle.com/datasets/ahmettyilmazz/fuel-consumption.
